# Characterization and comparison of gene-centered human interactomes

**DOI:** 10.1093/bib/bbab153

**Published:** 2021-05-19

**Authors:** Ettore Mosca, Matteo Bersanelli, Tommaso Matteuzzi, Noemi Di Nanni, Gastone Castellani, Luciano Milanesi, Daniel Remondini

**Affiliations:** Institute of Biomedical Technologies, National Research Council, Segrate (Milan), 20090, Italy; Humanitas University, Department of Biomedical Sciences, Pieve Emanuele (Milan), 20090, Italy; Department of Physics and Astronomy, University of Bologna, Bologna, 40127, Italy; Institute of Biomedical Technologies, National Research Council, Segrate (Milan), 20090, Italy; Department of Experimental, Diagnostic and Specialty Medicine, University of Bologna, Bologna, 40127, Italy; Institute of Biomedical Technologies, National Research Council, Segrate (Milan), 20090, Italy; Department of Physics and Astronomy, University of Bologna, Bologna, 40127, Italy

**Keywords:** network biology, molecular interactions, interactome, comparison

## Abstract

The complex web of macromolecular interactions occurring within cells—the interactome—is the backbone of an increasing number of studies, but a clear consensus on the exact structure of this network is still lacking. Different genome-scale maps of human interactome have been obtained through several experimental techniques and functional analyses. Moreover, these maps can be enriched through literature-mining approaches, and different combinations of various ‘source’ databases have been used in the literature. It is therefore unclear to which extent the various interactomes yield similar results when used in the context of interactome-based approaches in network biology. We compared a comprehensive list of human interactomes on the basis of topology, protein complexes, molecular pathways, pathway cross-talk and disease gene prediction. In a general context of relevant heterogeneity, our study provides a series of qualitative and quantitative parameters that describe the state of the art of human interactomes and guidelines for selecting interactomes in future applications.

## Introduction

Biological processes take place through the dynamic interaction of different types of molecular entities within highly organized environments. The characterization of the complex web of macromolecular interactions occurring within human cells, the interactome, is an essential task to explain the genetic architecture of complex diseases [1]. The interactome is being used in several approaches as a map to guide our understanding of how alterations perturb the system as a whole [2, 3]. Such interactome-based (or more generally network-based) methods have been developed to solve problems in all three broad categories of integrative analyses [4], namely (i) to understand molecular behaviors, (ii) to find disease subtypes and (iii) to predict an outcome or phenotype. Indeed, the interactome represents a powerful framework to understand and integrate omics datasets [5–9]. In these approaches, molecular interactions are used to capture systems-level patterns (e.g. active network regions, disease modules) that go beyond the knowledge attainable by analyzing each individual perturbation (e.g. mutation, expression change) separately from the others (i.e. as if they affect the phenotype by acting independently) [2].

In contrast to human genome and transcriptome, a unique reference model is not available for the interactome, which is still far from completeness. For example, while the first reference map for human metabolism has been produced [10], the one involving protein–protein interactions (PPIs) is still in progress [11]. At present, different reconstructions of the gene-centered human interactome are available. In these interactomes, nodes are genes while edges represent different types of interactions involving genes and gene products. This representation simplifies the many types of players involved (e.g. DNA sequence, protein isoforms) and interactions (PPI, protein-DNA) involved, providing a useful model to integrate many other data types that are imputable to genes, like the scores (e.g. *P*-values, fold-changes, etc.) emerging from omics data analysis. A node represents the gene itself or any of its products, while edges accommodate both biophysical (direct) and functional (indirect) interactions. In gene-centered interactomes, biophysical interactions mainly include PPI and protein–DNA interactions (PDI). Therefore, a PPI between genes *A* and *B* represents any PPI between any pair of products of the two genes, while a PDI between *A* and *B* indicates the binding between any protein encoded by *A* to gene *B*. Functional interactions represent any type of biological relation between two genes that do not involve a direct contact, for example co-expression relations, genetic interactions and links between enzymes that catalyze adjacent reactions in metabolic pathways.

Gene-centered interactomes differ in terms of type of interaction included, data sources and assembling procedure. We can distinguish three classes of interactomes: high-throughput biophysical (‘HTBP’) interactomes, ‘integrative’ interactomes and ‘integrative–predictive’ interactomes. HTBP interactomes are the state of the art in terms of reconstructing the interactome in a biological model (e.g. cell lines) detecting PPIs by means of a high-throughput assay, like the yeast two hybrid screening, affinity purification followed by mass spectrometry and co-fractionation [11]. Integrative interactomes collect interaction data from both primary databases and meta-databases. Primary databases collect experimental data from both small- and large-scale studies, while meta-databases integrate and unify interactions from multiple primary databases. Integrative–predictive interactomes contain interactions collected from multiple sources as well as predicted interactions, hypothesized on the basis of a series of evidences, like co-expression, co-participation in molecular pathways and co-occurrence in scientific publications.

In such a heterogeneous and evolving scenario, which lacks a reference model, it is not trivial to decide which interactome or set of interactomes is the most appropriate for a particular application (e.g. disease gene prediction). In order to guarantee a good coverage of the totality of the genes, it is common to perform network-based analysis using interactomes defined combining multiple sources. In some works, the results obtained using different interactomes on the same data are compared assessing the variation of the studied outcome (e.g. [12, 13]) or joined in a consensus (e.g., [14]). However, quite often, a single interactome is used.

Recently, a benchmark for the performance of several interactomes on a particular task, namely disease prioritization, found that the choice of interactome matters greatly [15]. Here, we characterized 19 interactomes on the basis of topological properties, protein complexes, molecular pathways, pathway cross-talk (PCT) and performance in disease gene prediction. Our study describes the state of the art of the most general purpose, complete and widely used interactomes, and offers a series of hints to guide the choice of interactomes in future applications.

## Material and Methods

### Interactome collection and harmonization

The original genes/protein identifiers chosen by the authors of each interactome (Entrez Gene, gene symbols, Uniprot, Ensembl transcript, Ensemble gene, Ensemble protein, iRefIndex icrogid) were mapped to Entrez gene identifiers. Mappings between Entrez Gene identifiers and other identifiers were collected from Entrez Gene FTP site ftp://ftp.ncbi.nih.gov/gene (26 February 2019), Uniprot FTP site https://www.uniprot.org/downloads (26 February 2019), R package biomaRt [16] (26 February 2019) and (where available) by the authors of the interactomes (STRING [17] https://string-db.org/mapping_files/entrez, iRefIndex [18] https://irefindex.vib.be/wiki/index.php/Protein_identifier_mapping). Some interactomes included a minor number of interactions involving identifiers from other (non-human) species. Only interactions between human genes were considered. All interactomes included a major component, the so-called largest connected component (LCC), which involved more than the 99% (median value) of the total genes of the interactome, and a few minor components: only the LCCs were considered in our study. Details about the number of genes and interactions are provided in Supplementary Table S1 available online at https://academic.oup.com/bib and source of interactions in Supplementary Table S2 available online at https://academic.oup.com/bib.

The two interactomes S04T and S07T, derived from STRING, were obtained selecting only the links with confidence score $\ge$0.4 and $\ge$0.7, respectively, since these are two typical thresholds used for this database to identify significant relationships. The other two interactomes derived from STRING, S04 and S07, were obtained recalculating the confidence score excluding the contribution of text mining, by means of the script provided at URL http://string-db.org/download/combine_subscores.py (see https://string-db.org/cgi/help.pl). When multiple pairs of Ensembl protein identifiers, characterized by different STRING confidence scores, mapped to the same pair of Entrez gene identifiers, the highest score was considered as representative of the interaction between the two genes.

iRefIndex complexes were transformed into a list of binary interactions following the so-called spoke model (interactions occur only between the ‘bait’ protein and each of the others) if the bait protein was indicated, and, otherwise, to the matrix model (all-pairs interactions) (see https://irefindex.vib.be/wiki/index.php/README_MITAB2.6_for_iRefIndex_15.0).

### Topological analysis

Topological analysis was performed using the R package igraph [19]. The overall degree distribution of each interactome was fitted with a power law distribution using the method proposed by Clauset *et al*. [20] (implemented in the R package poweRlaw [21]), which jointly estimates the power law exponent and the power law onset threshold *K*_min_, i.e. the degree above which the distribution is a power law (see Supplementary Methods available online at https://academic.oup.com/bib for further details). Goodness-of-fit was assessed by a semi-parametric bootstrap procedure. The *P*-value was defined as the fraction of times the KS statistics of the fit of the synthetic distributions (bootstrap) is greater than that for the empirical data fit. Therefore, a low *P*-value indicates rejections of the power law hypothesis.

The plausibility of scale-free hypothesis was tested according to several criteria proposed in a recent comprehensive survey on scale-free networks [22]. Since our interactomes are all undirected networks, we introduced a simplified version of the taxonomy proposed therein. We stratified interactomes in four different levels of plausibility for the scale-free distribution hypothesis:

(i) None: Interactomes for which the semi-parametric bootstrap has a *P*-value lower than 0.1, showing that the power law must be rejected.
(ii) Weak: Interactomes such that power law distribution cannot be rejected, i.e. semi-parametric bootstrap has a *P*-value greater than 0.1, and such that the fitted tail (data points ${x}_i>{K}_{\mathrm{min}}$) contains at least 200 genes.
(iii) Medium: Interactomes that satisfy weak constraints and either (a) have power law exponent in the range 2 < α < 3 or (b) such that power law fits better than exponential or lognormal distribution in the same degree range.
(iv) Strong: Interactomes satisfying both medium constraints.

To compare global measures of interactomes with those of a scale-free model, 10 instances of the Barabasi–Alberts (BA) model were generated, for each interactome, using the same number of genes and links of the considered interactome.

### Biological pathways, protein complexes and disease-associated genes

Pathways and protein complex definitions were derived from the NCBI Biosystems database [23], considering KEGG [24], Reactome [25] and GO [26]. In addition, protein complexes were collected from CORUM [27]. Pathways were filtered to keep those composed of a minimum of 10 genes and a maximum of 500 genes. Protein complexes were filtered to keep those composed of a minimum of 3 genes and a maximum of 500 genes. Genes associated with cancer were collected from Cosmic [28], while those associated with ‘Ataxias, Hereditary’ (ATX), ‘Autistic Disorder’ (ASDs), ‘Rheumatoid Arthritis’ (RA) and ‘Parkinson Disease’ (PD) were collected from DisGeNET [29] (Supplementary Table S3 available online at https://academic.oup.com/bib).

### Connected component fraction

Given a gene set *S* and its subset ${S}_C\subseteq S$, defined by the genes in *S* that are connected to at least another gene in *S* in the considered interactome, the connected component fraction (CCF) is as follows:

$$ \mathrm{CCF}=\left|{S}_C\right|\!\left/ \!\left|S\right|\right.. $$

### Network diffusion

Given an input gene list *L* and a gene network encoded as the *n*-by-*n* symmetrically normalized adjacency matrix $\boldsymbol{W}={\boldsymbol{D}}^{-1/2}\boldsymbol{A}{\boldsymbol{D}}^{-1/2}$, obtained as previously described [12, 30, 8], the *n*-sized vector ${\boldsymbol{X}}_0$ was defined as a binary vector with elements equal to 1 for the genes in *L* (e.g. disease genes), and null values for all the other genes. Network diffusion finds the vector ${\boldsymbol{X}}^{\ast }$ in which the quantities initially available in ${\boldsymbol{X}}_0$ are subject to smoothing according to the pattern of interactions $\boldsymbol{W}$. The vector ${\boldsymbol{X}}^{\ast }$ was calculated using the iterative procedure:

$$ {\boldsymbol{X}}_{t+1}=\alpha \boldsymbol{W}{\boldsymbol{X}}_t+\left(1-\alpha \right){\boldsymbol{X}}_0 $$

$$ {\boldsymbol{X}}^{\ast }=\underset{t\to \infty }{\lim }{\boldsymbol{X}}_t $$

where $\alpha$ (here set to 0.7 as in previous works [30]) is a parameter that weights to which extent the initial information is retained or spread throughout the network.

### Pathway cross-talk

To quantify the PCT between two pathways (*P*_1_, *P*_2_), each composed by a series of genes *g_i_*, the score *S_12_* was defined as the average network proximity of ${P}_2$ genes from ${P}_1$ in a given interactome *I*:

$$ {S}_{12}=\frac{\sum_{g_i\in{P}_2}{x}_i^{\ast \prime }}{\mid{P}_2\mid } $$

where ${x_i}^{\ast \prime}\in{\boldsymbol{X}}^{\ast \prime }$ is the normalized network proximity value of the gene *g_i_*. The normalized network diffusion vector ${\boldsymbol{X}}^{\ast \prime }$ was obtained through network diffusion of the source vector ${\boldsymbol{X}}_0$, in which ${P}_1$ genes are set equal to 1 and other genes to 0, and thus enables the direct comparison between different pathways:

$$ {\boldsymbol{X}}^{\ast \prime }={\left[\sum_i{\boldsymbol{x}}_{\boldsymbol{i}}^{\ast}\right]}^{-1}{\boldsymbol{X}}^{\ast } $$

In other words, ${S}_{12}$ quantifies the average proportion of an hypothetical substance that is found at steady state in ${P}_2$ genes, after a network diffusion process in which the substance enters in the network $I$ through ${P}_1$ genes. Because the two pathways correspond to two different subnetworks of interactome *I*, the calculation of *S_21_*, the average network proximity of ${P}_1$ genes from ${P}_2$ genes, yields a numerically different result. We therefore defined PCT between ${P}_1$ and ${P}_2$ as the average between ${S}_{12}$ and ${S}_{21}$:

$$ \mathrm{PCT}\left({P}_1,{P}_2\right)=\frac{S_{12}+{S}_{21}}{2} $$

The higher the PCT the shorter the paths among the genes of the two pathways in *I*.

### Performance assessment in recovering known disease genes

For each interactome and for each studied disease, the performance in recovering known disease genes was assessed by means of 5-fold cross-validation. Network diffusion was used to obtain genome-wide gene prioritizations starting from a pool of known disease genes. In each trial *i*, a random sample of $4/5$ of the known disease genes was used to initialize the input vector (set to 1 in ${\boldsymbol{X}}_0$). The performance in prioritizing the remaining one-fifth of the known disease genes was assessed calculating the partial area under the receiver operating characteristic curve (pAUC) at 20% of false-positive rate (*f*):

$$ \mathrm{pAUC}={\int}_0^{0.2}\mathrm{ROC}(f)\mathrm{d}f $$

The performance value used for each (interactome, disease) pair was the average pAUC over five trials.

### Correlation analysis

Unless stated otherwise, correlations were calculated using non-parametric Spearman rank-based correlation coefficient. The set of interactome-by-interactome correlation matrices obtained in each analysis type, i.e. topology (T), protein complexes (PC), PATHways (PATH), PCT and disease gene prioritization (DGP), was summarized into five (one per type) interactome-by-interactome correlation matrices, ***C***_T_, ***C***_PC_*, **C***_PATH_, ***C***_PCT_ and ***C***_DGP_:

$$ {\boldsymbol{C}}_t=1\!\left/ \!{n}_t\right.{\sum}_j^{n_t}{\boldsymbol{C}}_j $$

where *t* = {T, PC, PATH, PCT, DGP} and *n_t_* is the number of correlation matrices generated for each analysis type *t*. In particular, *n_t_* was equal to: 4, in topological analysis (different types of centrality measures); 3, CCF analysis of protein complexes (GO, KEGG and CORUM); 3, in pathway CCF analysis (GO, KEGG and Reactome); 5, in disease gene prioritization (ATX, ASDs, Cancer, PD and RA); 1, in the analysis of PCT (KEGG), due to the computational burden of this analysis type.

The overall similarity network among interactomes was defined considering the weight matrix ${\boldsymbol{C}}_I=\sum_t{C}_t$ and, for each interactome, only its top four most similar interactomes. The community structure was assessed using the fast greedy modularity optimization algorithm [31] implemented in igraph [19].

The aggregate correlation of an interactome *i* with all the others, ${R}_i$, was defined as the sum of the average correlations between interactome *i* and the other interactomes:

$$ {R}_i=\sum_t\frac{\sum_{j\ne i}{c}_{ijt}}{n-1} $$

where ${c}_{ijt}$ is an element of matrix ***C****_t_* and *n* is the number of interactomes.

## Results

**Figure 1:**
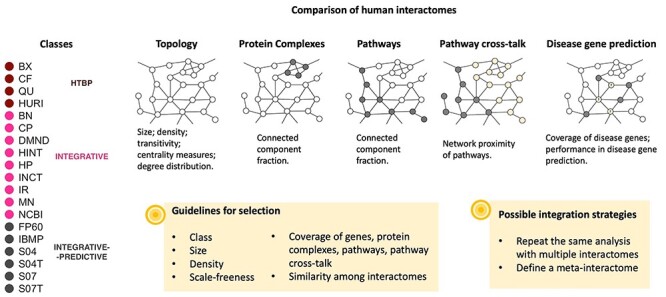
Characterization of human interactomes. We considered topological properties, protein complexes, molecular pathways, PCT and performance in disease gene prediction. Our study describes the state of the art and offers a series of hints to guide the choice of interactomes in future applications.

We studied a total of 19 popular interactomes spanning all the three broad classes: ‘HTBP’ [32–35], ‘integrative’ [18, 36–43] and ‘integrative–predictive’ [17, 44, 45] (Figure 1, Table 1; Supplementary Table S1 available online at https://academic.oup.com/bib). We included four variants of STRING (S04, S04T, S07 and S07T) to explore the effect of different ways of selecting links in such popular resource.

**Table 1:** Gene-centered human interactomes

ID	Name (version)	Class	No. of interactions	No. of genes
BX	Bioplex (4a) [29]	HTBP	56 401	10 880
CF	Cofrac15 [30]	HTBP	15 513	3191
HURI	HURI [8]	HTBP^a^	27 084	8029
QU	QUBIC [31]	HTBP	14 696	4379
BN	Biana (guildify 2.0) [32]	Integrative	339 698	13 246
HINT	HINT (April 2019) [33]	Integrative	164 255	14 372
HP	HIPPIE (2.2) [34]	Integrative	404 020	18 038
INCT	Intact (2019_07_03) [35]	Integrative	174 388	15 539
IR	irefindex (15.0) [18]	Integrative	476 437	17 522
DMND	DMND [36]	Integrative	138 045	13 244
NCBI	NCBI (15/09/2017) [37]	Integrative	326 859	17 655
CP	ConsensusPathDB (guildify 2.0) [38]	Integrative	273 005	16 066
MN	MULTINET [39]	Integrative	105 573	13 387
FP60	FPCLASS [40]	Integrative–predictive	258 107	10 403
IBMP	InBio_web (core 2019_02_26) [41]	Integrative–predictive	652 636	17 458
S04	String, CS > 0.4 (11) [17]	Integrative–predictive	490 587	15 800
S04T	String including TM, CS > 0.4 (11) [17]	Integrative–predictive	986 054	18 863
S07	String, CS > 0.7 (11) [17]	Integrative–predictive	357 054	12 747
S07T	String including TM, CS > 0.7 (11) [17]	Integrative–predictive	417 012	16 721

HTBP, high-throughput biophysical interactome; CS, confidence score; TM, text mining.

^a^The interactome contains a minor number of biophysical interactions manually curated from small-scale studies.

First of all, we characterized each interactome by a series of topological measures of local and global nature, including an assessment of the ‘scale-freeness’. Then, we studied to which extent interactomes capture known protein complexes and molecular pathways. We also characterized interactomes on the basis of the relations among pathways (PCT) and a common task, disease gene prediction. In each of such analyses, we assessed the correlation between interactomes. Our characterization of interactomes offers a description of the state of the art and a series of criteria that can be used as guidelines for interactome choice or integration strategy in future applications (Table 2).

**Table 2:** Interactome classification

ID	class	Size (Figure 2)	Density (Figure 2)	Scale freeness (Figure 2)	Coverage		Disease gene prediction (Figure 6)	Top 2 highly correlated interactomes					Similarity cluster (Figure 7)	R (Figure 7)
					Protein Complexes (Figure 3)	Pathways (Figure 4)		Topology (Supplementary Table S5)	Protein Complexes (Supplementary Table S5)	Pathways (Supplementary Table S5)	PCT (Supplementary Table S5)	Disease gene prediction (Supplementary Table S5)		
BX	HTBP	***	*	Strong	*	**	*	NCBI;HP	HINT;NCBI	HINT;BN	QU;HURI	NCBI;HP	2	*
CF	HTBP	*	***	Strong	*	*	**	BN;HINT	BN;BX	BN;HINT	QU;HINT	HINT;BN	3	*
HURI	HTBP*	**	*	Medium	**	**	**	DMND;BN	BN;MN	INCT;CP	CP;DMND	BN;DMND	3	***
QU	HTBP	*	*	Medium	*	*	*	HINT;INCT	INCT;BN	BN;INCT	BX;HURI	HINT;INCT	3	*
BN	INTGR	****	****	None	***	***	*	CP;HINT	HINT;NCBI	HINT;INCT	HINT;CP	CP;HINT	3	****
HINT	INTGR	****	*	Weak	****	****	**	CP;IR	NCBI;CP	BN;CP	CP;NCBI	CP;NCBI	3	*****
HP	INTGR	*****	**	Weak	****	*****	***	NCBI;CP	NCBI;CP	NCBI;CP	NCBI;CP	NCBI;CP	2a	****
INCT	INTGR	****	*	None	***	****	**	CP;HINT	HINT;CP	HINT;CP	HP;CP	CP;HP	3	***
IR	INTGR	*****	***	Medium	*****	*****	***	CP;HP	HP;NCBI	HP;NCBI	HP;CP	HP;CP	2	*****
DMND	INTGR	****	*	Medium	****	*****	**	CP;BN	NCBI;FP60	NCBI;CP	HURI;FP60	CP;HP	2	****
NCBI	INTGR	*****	**	Medium	****	*****	**	HP;CP	CP;HP	CP;HP	HP;CP	HP;CP	2a	*****
CP	INTGR	*****	**	Weak	****	*****	**	HP;NCBI	NCBI;HP	NCBI;HP	NCBI;HP	HP;NCBI	2a	*****
MN	INTGR	****	*	Medium	****	****	**	DMND;CP	DMND;FP60	DMND;NCBI	HURI;S04	DMND;CP	2	***
FP60	INTGR-PRED	***	*****	None	****	****	**	DMND;CP	DMND;MN	CP;NCBI	HURI;DMND	DMND;CP	2	***
IBMP	INTGR-PRED	*****	****	None	****	*****	**	CP;HP	HP;IR	CP;HP	CP;HP	CP;IR	2	*****
S04	INTGR-PRED	*****	****	Weak	*****	*****	***	S07;S07T	S07T;S07	S07;S07T	S07;S07T	S07;S04T	1	***
S04T	INTGR-PRED	*****	*****	Weak	*****	*****	*****	S07T;S04	S04;S07T	S07T;S04	S07T;S04	S07T;S07	1	**
S07	INTGR-PRED	****	****	None	*****	*****	***	S07T;S04	S04;S07T	S04;S07T	S07T;S04	S07T;S04T	1	***
S07T	INTGR-PRED	*****	***	None	*****	*****	****	S07;S04	S04;S07	S04;S07	S07;S04	S07;S04T	1	***

Asterisks indicate the interval of values to which interactomes belong. See the corresponding figure or table for further details.

### Topological properties

**Figure 2:**
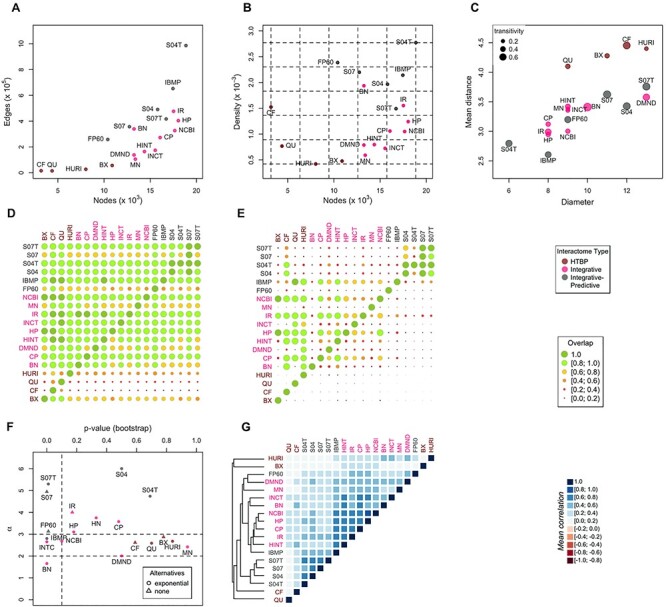
Topological properties of 19 human interactomes. (**A**) Number of interactions versus number of genes (size). (**B**) Density versus size. (**C**) Diameter versus mean distance; dot size is proportional to the mean gene transitivity (or clustering coefficient, i.e. the fraction of closed triangles in the network). (**D**) Overlap between genes, defined as the ratio between the genes shared by each couple of interactomes and the size of the interactome in the corresponding column label; this implies that a row indicates to which extent the interactome (row label) includes other interactomes, while a column indicates to which extent the interactome (column label) is included in other interactomes. (**E**) Interaction overlap, defined analogously to gene overlap. (**F**) Assessment of the scale-free hypothesis: power law exponent (alpha) and *P*-value; circles (exponential): the exponential distribution fits better than power law; triangles (none): power law fits better than other distributions. (**G**) Average correlation values of topological measures; the dendrogram was obtained by complete linkage method.

The 19 interactomes show relevant variations in terms of genes and interactions, not only between classes, as expected by the different designing principles, but also within the same class (Figure 2A). For example, HTBP interactomes contain a number of genes ranging approximately from 3000 to 11 000; a number, this latter, comparable with that of FP60, the smallest interactome of integrative–predictive class. Integrative and integrative–predictive interactomes are comparable in terms of gene number (from 11 000 to 19 000), but integrative–predictive interactomes have a higher link density: in these interactomes, the average number of links per node is in the interval [20, 49], while it spans [10, 30] in integrative interactomes and [3, 5] in HTBP interactomes (Figure 2B).

From the analysis of global measures, that is, diameter, mean distance between nodes and the mean clustering coefficient (or transitivity), we can draw the following picture: as expected, mean distance and diameter are correlated; moreover, higher density is associated with lower mean distance and higher clustering, with HTBP class on one side and ST04 on the other (Figure 2C). Clustering coefficient is lower than 0.1 for most of the interactomes with less than 24 links per node on average (with the exception of CF and DMND), while for the others it is in the range 0.2–0.7 (Supplementary Figure S1 available online at https://academic.oup.com/bib).

As expected, integrative interactomes that share interaction sources (e.g. NCBI and HP, see Supplementary Table S2 available online at https://academic.oup.com/bib) have many links and genes in common; on the other side, HTBP interactomes, due to their independent derivation and different experimental techniques, have a small mutual overlap [11] (Figure 2D and E).

To further explore the ‘scale-freeness’ of interactomes, we compared the values of global measures in interactomes with those of scale-free networks (BA models) of similar density and assessed the strength of the evidences in favor of the scale-free hypothesis. The mean distances and clustering coefficients of interactomes are always higher than those of BA nets (Supplementary Figure S2 available online at https://academic.oup.com/bib). Moreover, the clustering coefficient of interactomes is more variable, from 0.1 to 0.7, than BA networks, where it is almost constant (Supplementary Figure S2 available online at https://academic.oup.com/bib). Overall, for most interactomes, the shape of the degree distribution shows a power law-like trend in an intermediate range of values (which depends on network size and density), with a deviation in the low-degree and right-tail part of the distribution as observed for many real-world nets [46] (Supplementary Figure S3 available online at https://academic.oup.com/bib). More in detail, for six interactomes of the integrative and integrative–predictive classes, the power law hypothesis must be rejected (*P* < 0.1) (Figure 2F), while all other interactomes show at least weak evidence of scale freeness (Figure 2F, Supplementary Table S4 available online at https://academic.oup.com/bib). Strong evidence (i.e. the power law fitting better than other distributions, and an exponent plausible with other real scale-free networks) is satisfied only by two HTBP interactomes: CF and BX. Also HURI, DMND, MN, NCBI and QU have a plausible exponent in the range [2, 3], but the goodness of exponential fitting is higher. IR does not have exponent in the interval [2, 3], but the power law is the most likely fit distribution. The two STRING variants with confidence score equal to 0.4 show weak evidence, while the other integrative–predictive interactomes none.

Highly connected genes (hubs) have proved to play relevant roles in physiological and pathological conditions [46]. We analyzed to which extent interactome share the same hubs. To do so, we considered the genes in the top two percentiles (right tail) of the degree distribution and found that the overlap among hubs is significantly higher than that observed in permuted versions of the same degree distributions (Supplementary Figure S4A available online at https://academic.oup.com/bib). Quantitatively, the number of shared hubs between at least two interactomes is around 900; this number drops to 32 when considering at least 12 interactomes, while no hubs are shared by more than 16 interactomes (Supplementary Figure S4B available online at https://academic.oup.com/bib), reflecting differences and complementarity in the interactome panorama. The most recurrent hub is the histone deacetylase 1 (*HDAC1*, <*d*> = 360), which is included in the first two percentiles of 16 interactomes and is available in all of them, followed by E1A-binding protein p200 (*EP300*, <*d*> = 542.5), *BRCA1* DNA repair associated (*BRCA1*, <*d*> = 376), heat shock protein 90 alpha family class A member 1 (*HSP90AA1*, <*d*> = 433), tumor protein p53 (*TP53*, <*d*> = 553) and heat shock protein family A (Hsp70) member 8 (*HSPA8*, <*d*> = 433), which appear in at least 17 interactomes and in the top two percentiles of 15 interactomes (Table 3). Interestingly, the median degree of a hub tends to increase with the number of interactomes in which the hub appears (Supplementary Figure S4C available online at https://academic.oup.com/bib); in other words, hub genes with a higher degree tend to be more shared than hubs with lower degree.

**Table 3:** The most recurrent hubs

Symbol	Description	Availability	Median (d)	SD (d)	Occurrence as hub
*HDAC1*	Histone deacetylase 1	19	360	275.6	16
*EP300*	E1A binding protein p300	18	542.5	317.5	15
*BRCA1*	BRCA1 DNA repair associated	17	376	321.6	15
*HSP90AA1*	Heat shock protein 90 alpha family class A member 1	19	433	457.2	15
*TP53*	Tumor protein p53	17	553	502.3	15
*HSPA8*	Heat shock protein family A (Hsp70) member 8	19	433	553.5	15
*UBE2I*	Ubiquitin conjugating enzyme E2 I	19	312	211.5	14
*PPP2R1A*	Protein phosphatase 2 scaffold subunit A alpha	19	324	276.2	14
*CREBBP*	CREB binding protein	18	389.5	278.2	14
*CTNNB1*	Catenin beta 1	19	363	309.7	14
*SRC*	SRC proto-oncogene, non-receptor tyrosine kinase	17	438	297.0	14
*EGFR*	Epidermal growth factor receptor	17	482	483.0	14
*RPS3*	Ribosomal protein S3	19	296	215.1	13
*RPS2*	Ribosomal protein S2	18	291	210.5	13
*PLK1*	Polo like kinase 1	19	269	218.8	13
*H2AFX*	H2A histone family member X	18	286	224.8	13
*MAPK1*	Mitogen-activated protein kinase 1	18	351	302.6	13
*YWHAZ*	Tyrosine 3-monooxygenase/tryptophan 5-monooxygenase activation protein zeta	19	356	345.6	13
*GRB2*	Growth factor receptor bound protein 2	18	591	370.9	13
*JUN*	Jun proto-oncogene, AP-1 transcription factor subunit	18	325	567.9	13
*RPS8*	Ribosomal protein S8	18	298.5	176.2	12
*RPS3A*	Ribosomal protein S3A	19	277	194.7	12
*PIK3R1*	Phosphoinositide-3-kinase regulatory subunit 1	18	284	236.0	12
*NPM1*	Nucleophosmin 1	19	324	277.8	12
*CDK1*	Cyclin-dependent kinase 1	19	301	297.6	12
*MDM2*	MDM2 proto-oncogene	17	186	281.6	12
*CDC5L*	Cell division cycle 5 like	19	573	301.8	12
*CDK2*	Cyclin dependent kinase 2	19	292	320.0	12
*HSPA5*	Heat shock protein family A (Hsp70) member 5	18	219.5	321.9	12
*ESR1*	Estrogen receptor 1	17	425	335.3	12
*MYC*	MYC proto-oncogene, bHLH transcription factor	18	483.5	491.1	12
*UBC*	Ubiquitin C	16	1036	2473.4	12

Genes belonging to the top two percentiles of the degree (d) distribution (hubs) of at least 12 interactomes. SD, standard deviation.

Finally, in order to test single-node similarities, we compared four centrality measures, which highlight different quantifications of node relevance: degree, the number of first neighbors; betweenness, the fraction of shortest paths passing through a node; closeness, the inverse of the average length of the shortest paths between the node and all other nodes in the graph; and spectral centrality (Pauls and Remondini 2012), which quantifies the importance of a node in relation to the deformation of the graph Laplacian. Apart from spectral centrality, the other three measures are highly correlated independently from the specific interactome topology (Supplementary Figures S5 and S6 available online at https://academic.oup.com/bib), thus conveying very similar information in terms of node ranking. We observed a similar distribution of correlation values (Spearman’s *r*_s_) (medians close to 0.43) for such three measures, while lower values (median of 0.15) for spectral centrality (Supplementary Figure S7 available online at https://academic.oup.com/bib).

We quantified the correlation of gene-level centrality scores among interactomes (Figure 2G, Supplementary Table S5 available online at https://academic.oup.com/bib). The correlation analysis of degree, betweenness and closeness revealed that the four variants of STRING form a group on their own. On the one hand, they show a high similarity among themselves, meaning that including text mining-derived interactions and varying confidence score did not affect significantly the local structure of the network: gene ranking by centrality is similar even if the links in S04 are twice as many than S07, an interesting observation since they have different global properties. On the other hand, they are much less correlated with other interactomes meaning that their local topology is different, even for interactomes of comparable size. Moreover, NCBI and HP are highly similar and can be included in a second group along with CP, IR, HINT. IBMP, despite being integrative-predictive, is closer to such a group, to which BN and INCT are also related. The integrative–predictive interactome FP60 forms a cluster with DMND and MN: this shows indeed a low overlap with other interactomes in terms of interactions. The four HTBP interactomes show very different intra-class centrality profiles, probably reflecting the differences between the experimental techniques used and the high number of false negatives (i.e. not identified interactions) of such techniques, as suggested by Luck *et al*. [11]. They are also poorly correlated with interactomes of other classes. Interestingly, the union of HTBP interactomes is more correlated with integrative interactomes than any single HTBP interactome (Supplementary Figure S6 available online at https://academic.oup.com/bib). We obtained a similar picture analyzing the centrality measures of the 1021 genes shared by all 19 interactomes (Supplementary Figure S8 available online at https://academic.oup.com/bib).

### Network representation of protein complexes and biological pathways

To assess how protein complexes and biological pathways are represented in the interactomes, we defined a simple score, the CCF. The higher the CCF the higher the number of protein complex (or pathway) members connected to each other.

**Figure 3:**
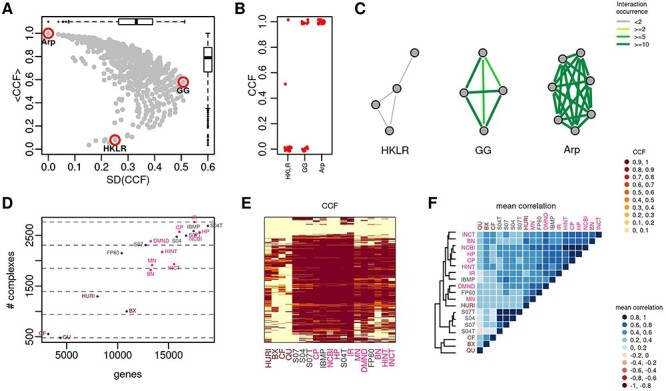
Protein complexes. (**A**) Average CCF (<CCF>) and standard deviation of CCF (SD(CCF)) across interactomes. (**B**) CCFs of three protein complexes: HOOK2-KCL3-LRGUK1-RIMBP3 (HKLR), GPI-GnT (GG) activity complex and Arp2/3 (Arp) complex. (**C**) Network visualization of three protein complexes with link colored by their occurrence in the interactomes. (**D**) Number of protein complexes (#) with CCF > 0.5 in relation to interactome size. (**E**) Heatmap of CCF values. (**F**) Average correlation values of protein complex CCFs; the dendrogram was obtained by complete linkage method.

Protein complexes tend to form topological modules within the interactome, that is locally dense subnetworks such that genes of a subnetwork tend to interact with other genes of the subnetwork rather than outside of it [47]. Since every protein complex member is expected to establish a PPI with at least another protein complex member, the CCF of protein complex is expected to be 1. We calculated the CCF of CORUM [13] protein complexes and found average values across interactomes that span from very low values, indicating poorly represented complexes, to very high values, standing for fully captured complexes, with a standard deviation up to 50% (Figure 3A, Supplementary Table S6 available online at https://academic.oup.com/bib). For example, none of the interactions among members of the HOOK2-KCL3-LRGUK1-RIMBP3 (HKLR) are reported in more than one interactome (Figure 3A–C). There are complexes for which there is a strong disagreement: an example is the GPI-GnT (GG) activity complex, for which the CCFs are equally distributed between the two extreme values of 0 (not even an interaction) and 1 (all proteins are connected) (Figure 3A–C). Lastly, we observed a series of complexes for which there is a strong consensus: an example is the Arp2/3 (Arp) complex, a major component of the actin cytoskeleton, where interaction occurs in more than 10 interactomes (Figure 3A–C).

Integrative interactomes represent from ~1800 to ~2800 protein complexes (out of a total of ~3000), with more than half of the proteins connected (CCF > 0.5) (Figure 3D and E). Despite the four HTBP interactomes capture a lower number of complexes (from ~600 to ~1300) (Figure 3D and E), they are complementary (Figure 3E): if taken together, they represent ~1800 complexes.

We assessed the correlation (Spearman) of protein complex CCFs among all pairs of interactomes and found that they are all positives with a median of ~0.5 (Supplementary Table S5 and Supplementary Figures S9 and S10 available online at https://academic.oup.com/bib). We observed similar results considering GO cellular components and KEGG structural components as sources for the protein complex definition (Supplementary Figures S9 and S10 available online at https://academic.oup.com/bib). Excluding minor differences, the similarity among interactomes in terms of CFF of complexes reflects what we found in the analysis of topological features (Figure 3F).

**Figure 4:**
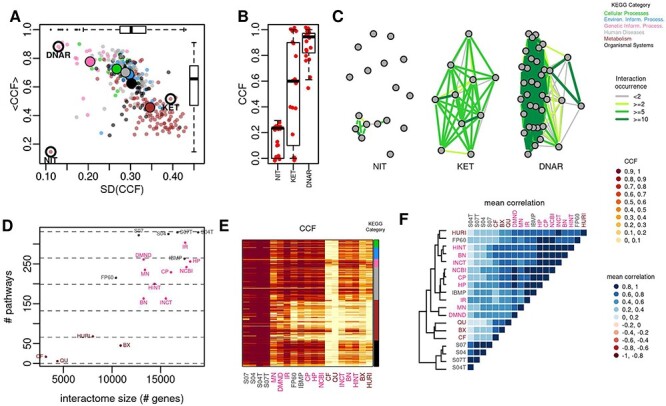
Molecular pathways. (**A**) Average CCF (<CCF>) and standard deviation of CCF (SD(CCF)) across interactomes; big dots represent median values calculated for each pathway category. (**B**) CCFs of three molecular pathways: nitrogen metabolism (NIT), synthesis and degradation of ketone bodies (KET) and DNA replication (DNAR). (**C**) Network visualization of three molecular pathways with link colored by their occurrence in the interactomes. (**D**) Number of molecular pathways (#) with CCF > 0.5 in relation to interactome size. (**E**) Heatmap of CCF values. (**F**) Average correlation values of pathway CCFs; the dendrogram was obtained by complete linkage method.

Unlike protein complexes, pathway members may or may not form topological modules. However, pathway members are expected to lie in network proximity within the interactome, forming a functional module, that is a group of genes that interact to fulfill a particular function [3]. Among KEGG pathways [22], we observed that CCF values vary by pathway category. In particular, metabolic pathways assume the lowest CCF values (<CCF> ~0.36), while those involved in genetic information processing have the highest values (<CCF> ~0.75) and are less variable (Figure 4A) (Supplementary Table S7 available online at https://academic.oup.com/bib). An example of a pathway with a low CCF is nitrogen metabolism (NIT) (Figure 4A–C), for which only a few links occur in more than 2 interactomes and only one link in more than 10 interactomes. An example of a pathway with a marked disagreement among interactomes is the synthesis and degradation of ketone bodies (KET): CCFs are distributed between 0 and 1, with a median of 0.4 (Figure 4A–C). On the other hand, DNA replication is the pathway associated with the highest CCF and a strong consensus (Figure 4A–C). STRING interactomes cover almost all pathways with CCF > 0.5 (Figure 4D and E). The other integrative interactomes form two groups (Figure 4D and E): one with a higher coverage and higher CCF values and the other with lower CCF and coverage. Among the HTBP interactomes, we observed higher coverage and CCF values in BX and HURI (Figure 4D and E).

Considering correlation values of pathway CCFs between interactomes, we found that the majority of correlations assume positive values with median of 0.54, while negative correlations emerged in a few cases, when comparing STRING with other interactomes (Supplementary Figures S9 and S10 available online at https://academic.oup.com/bib). The correlation analysis on pathways obtained from GO and Reactome lead to similar results, but with a minor number of negative correlation (Supplementary Table S5 and Supplementary Figures S9 and S10 available online at https://academic.oup.com/bib). The similarity among interactomes in terms of CFF of pathways showed some differences compared to the previous ones: we observed some rearrangement involving integrative interactomes (e.g. HN is closer to BN), and FP60 is closer to HURI (Figure 4F).

### Pathway cross-talk

**Figure 5:**
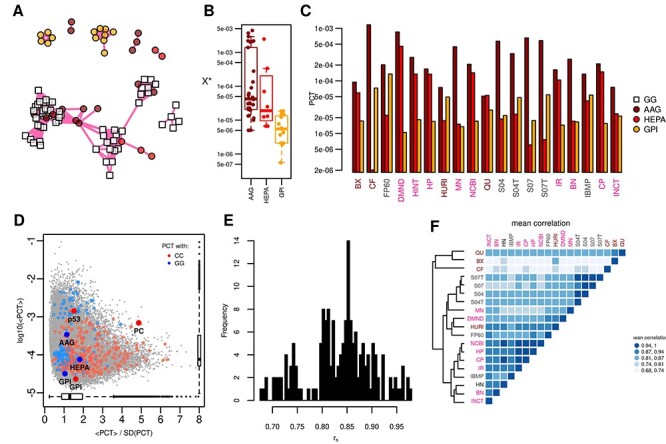
PCT. (**A**) Network representation of four pathways in GH interactome: GG, glycolysis/gluconeogenesis; AAG, alanine, aspartate and glutamate metabolism; HEPA, glycosaminoglycan biosynthesis—heparan sulfate/heparin; GPI, glycosylphosphatidylinositol GPI-anchor biosynthesis; only the interactions among the genes belonging to such pathways are shown. (**B**) Network proximity (X*) of genes of pathways AAG, HEPA and GPI from genes of pathway GG in DMN interactome. (**C**) PCT between GG–AAG, GG–HEPA and GG–GPI. (**D**) Average (<PCT>) and SNR of PCT between all-pairs pathways across interactomes. p53, p53 signalling; PC, pancreatic cancer. (**E**) Correlations (Spearman) of PCTs among interactomes. (**F**) Correlation values of PCTs; the dendrogram was obtained by complete linkage method.

In order to assess to which extent the relationships between molecular pathways are conserved across interactomes, we studied the PCT [23]. We defined the PCT as the average network proximity between the two sets of pathway genes, quantified by means of a network diffusion process [48]. Intuitively, the shorter the lengths of all-possible paths between the two sets of pathway genes, the closer the two pathways and the higher the PCT (Figure 5A). For example, the genes belonging to ‘glycolysis / gluconeogenesis’ (GG) and those belonging to ‘alanine, aspartate and glutamate metabolism’ (AAG) are close to each other in DMND interactome and have a higher PCT than that between GG and ‘Glycosaminoglycan biosynthesis—heparan sulfate/heparin’ (HEPA), or between GG and ‘Glycosylphosphatidylinositol GPI-anchor biosynthesis’ (GPI) (Figure 5A and B). The PCT of (GG, AAG) pair is higher than (GG, HEPA) and (GG, GPI) pairs in all interactomes (Figure 5C).

Throughout a distribution of average PCT (across interactomes) that spans five orders of magnitudes, we observed a median signal-to-noise ratio (SNR) of 1.3, with a right tail of conserved PCT with SNR up to 8 (Figure 5D; Supplementary Table S8 available online at https://academic.oup.com/bib). For example, the PCTs among cell cycle (CC) and other pathways are more conserved than those between GG and other pathways (Figure 5D). Among PCTs of CC, the one with p53 signaling (p53) is similar in magnitude to that between CC and pancreatic cancer (PC) but the latter is more conserved than the former (Figure 5D).

Globally, we found that PCTs of different interactomes are positively correlated (Figure 5E). The correlation between PCTs revealed similarities among interactomes that are closer to what we obtained analyzing the correlation of pathway CCFs (Figure 5F; Supplementary Table S5 available online at https://academic.oup.com/bib).

### Disease gene prioritization

Disease-gene prioritization is one of the main tasks for which interactomes are used [15, 49]. We studied the impact of using different interactomes for disease-gene prioritization. As a proof of principle, we considered five diseases (ATX, ASDs, Cancer, PD and RA) that differ in terms of the genes involved and with a sufficiently high number of associated genes to perform a cross-validation study in all interactomes.

Overall, the coverage of disease-associated genes is above 80% in integrative interactomes. In HTBP interactomes, the coverage decreases to 50% in HURI and BIOPLEX, and to 10% in COFRAC and QUBIC (Supplementary Table S9 available online at https://academic.oup.com/bib). Genes associated with cancer are more frequently included in interactomes than genes associated with the other diseases considered.

We obtained genome-wide gene prioritizations by means of network diffusion [48], a widely used approach [15, 49, 8] that follows the local hypothesis [2, 3] , that is the closer the proximity of a gene to known disease genes in the interactome, the higher the probability of gene–disease association (see Methods).

**Figure 6:**
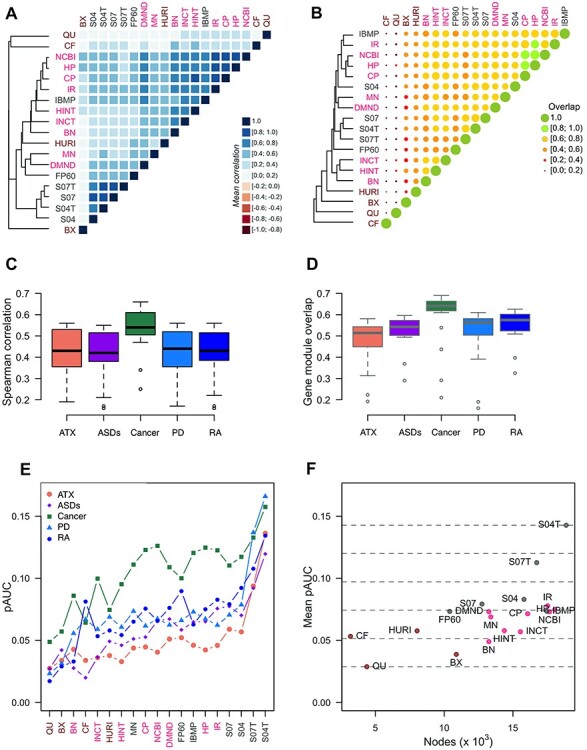
Disease gene prioritization. (**A**) Average correlation values between gene prioritizations. (**B**) Overlap of top ranking genes between interactomes. (**C**) Correlation of disease prioritization results between interactomes by disease. (**D**) Overlap of the top ranking genes between interactomes by disease. (**E**) Performance of each interactome estimated by means of 5-fold cross-validation; for clarity, lines have been added between the points; interactomes are ordered by decreasing average performance over all diseases considered (from right to left). (**F**) Average performance over the five diseases in relation to interactome size. (**A**, **B**) Dendrograms were obtained by complete linkage method.

We assessed the correlation of gene–disease association scores across interactomes (Supplementary Table S5 available online at https://academic.oup.com/bib). We obtained positive values (Spearman’s correlation) in all five diseases (Supplementary Figures S11 and S12), despite a high number of non-overlapping disease genes due to interactome-specific structural properties. The correlation between interactomes on the basis of full gene rankings yielded relations of similarity very close to those obtained by analyzing the topology (Figure 6A; Supplementary Figure S12 available online at https://academic.oup.com/bib). As gene prioritization is one of the main goals in disease module discovery, we also focused on the genes receiving the highest ranking in each interactome. The analysis of the overlap between the top ranking genes underlined relations of similarity differing from the previous ones: S04 is closer to the group of interactomes that includes NCBI, HP, CP, IR and IBMP; FP60 is farther from DMND and MN (Figure 6B). Whether considering full rankings or top ranks only, we found the highest similarity using cancer data (Figure 6C and D).

We assessed the performance of disease gene prioritization using 5-fold cross-validation and calculating the pAUC, which here reflects the recovery of a test set of known disease genes on the basis of a training set of disease genes. In almost all interactomes, cancer was the top ranking disease by pAUC, while ATX the worst (Figure 6E). STRING interactomes with text mining showed the highest performances, while IR and HURI were at the top of their class (Figure 6E). We remark that prioritization performance could not be simply explained by interactome size (Figure 6F).

### Overall similarity

**Figure 7:**
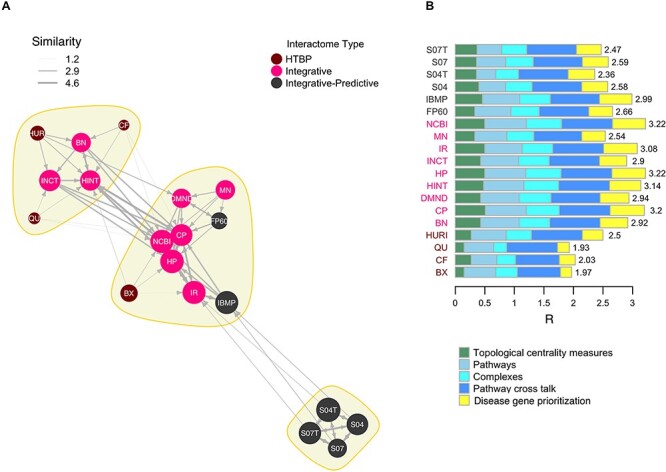
Overall interactome similarity. (**A**) Overall similarity network among interactomes; for each interactome, the arrows point to its four most similar interactomes (see Methods); yellow colored areas indicate community structure. (**B**) Aggregate correlation (R), decomposed in the contributions of each analysis type.

In order to summarize the similarities between interactomes, we defined a similarity network in which a link between two interactomes reflects the sum of their correlation coefficients resulting from the analysis of topology, protein complexes, pathways, PCT and disease gene prioritization (correlation matrix ${\boldsymbol{C}}_I$, Supplementary Table S5 available online at https://academic.oup.com/bib, see Methods). We found three communities (modularity = 0.34) of interactomes (Table 2, Figure 7A). The first community (#1) is defined by the four variants of STRING, which have high mutual correlations. The two STRING variants that do not include text mining are closer to IBMP (another integrative–predictive interactome), while those with text mining are more similar to IR. In the largest community (#2), we observed a sub-community formed by NCBI and HP and CP (#2a), a trio of highly correlated integrative interactomes. BN, HINT and INCT were assigned to the community (#3), which also includes three HTBP interactomes.

In addition, to further reduce the relations of similarity in a unique quantity (*R_i_*) per interactome, we aggregated the correlation coefficients (see Material and Methods, Supplementary Table S5 available online at https://academic.oup.com/bib). The higher the *R_i_*, the higher the correlation of the interactome with all the others (Table 2, Figure 7B). Members of group (#2b), as well as IR, obtained the highest values. IBMP and HURI scored first among, respectively, integrative–predictive and HTBP interactomes.

## Discussion

Currently available models of the human interactomes are incomplete. Given the increasing importance of network-based analyses of omics datasets, we compared 19 interactomes, comprised within three main types: HTBP, integrative and integrative–predictive. We took into account different criteria for their characterization: topological measures (local and global), coverage of known protein complexes and molecular pathways, communication among pathways (PCT) and a typical prediction task, i.e. disease gene prioritization.

Interactomes are topologically heterogeneous. Such heterogeneity goes beyond interactome size or density and involves degree distribution shape and clustering coefficient. While the debate about the ‘scale-freeness’ of real molecular networks is still open [22], our analysis showed that for the majority of interactomes the evidence supporting the scale-free hypothesis is weak or medium, while strong evidence is associated with only two HTBP interactomes.

We found a significant overlap of hubs among interactomes (the top 2 percentiles in the right tails of the degree distributions), when compared to random interactomes with the same degree distributions. However, considering that the studied interactomes can be seen as models of the same underlying reality, the observed overlap might be considered not satisfactory and indicates some relevant discrepancies on genes that play the role of hubs. The observation that the most shared hubs tend to have higher degrees might reflect an association between the pathological relevance of a gene (e.g. key role in one or more diseases) and the amount of evidences (studies) supporting its interactions.

The analysis of the coverage of protein complexes in terms of CCFs revealed a quite high median value: about 80% of the maximum possible value. We also found, however, a relevant variability of about 35% (standard deviation). Despite integrative and integrative–predictive interactomes cover most of the CORUM protein complexes with high CCF values, there are a series of protein complexes that are poorly represented in all interactomes. Interestingly, the coverage of protein complexes is complemental among HTBP interactomes.

Overall, molecular pathways displayed lower CCF median values than complexes. This was expected, considering that members of pathways are not expected to form topological modules. We observed a clear difference between some pathway categories: pathways involved in ‘Genetic Information Processing’ showed the highest CCFs, while ‘Metabolic’ pathways had the lowest. This can be explained by observing that most pathways of the former type are mainly composed of well-studied protein complexes (e.g. ‘Proteasome’, ‘RNA polymerase’, ‘Mismatch repair’). On the contrary, metabolic pathways are mostly composed of enzymes: while some of them are known to form protein complexes, this evidence is not available for many others. Since this sparseness of metabolic pathway members would exclude them from computational analyses that require connected networks, the authors of some interactomes introduced functional links between enzymes that catalyze adjacent biochemical reactions (e.g. DMND).

The analysis of PCTs revealed relevant correlations among interactomes. This quantification revealed a striking (rank-based) similarity in the relative positioning of pathways, despite topological differences. Moreover, it underlined the strongest/weakest PCTs in current interactomes, as well as their degree of conservation. This knowledge can be useful for PCT-based approaches, like those that use PCT inhibition as a tool to develop synergistic drug combinations [50].

Disease gene prioritization performance showed reproducibility across interactomes when applied to cancer or ATX, which output the best and worst performance results. On the contrary, the performances obtained for the other three diseases (ASDs, PD and RA) resulted to be more interactome specific. It is beyond the scope of this article to deeply investigate the reasons of such performance variation in relation to the characteristics of the disease under analysis and the interactome used to carry out the predictions. Nevertheless, our results, in agreement with previous studies focused on disease genes [15, 49], reveal the complexity of choosing an interactome for a given activity such as predicting disease genes, in which the input dataset (e.g. the disease) makes a difference in determining which interactome performs the best.

The four versions of STRING, despite the differences in interaction type and confidence, kept a high similarity among themselves throughout all the analyses. This similarity reflects a specificity in local properties in comparison with other interactomes of similar type and size (i.e. FP60 and IBMP). NCBI and HP and CP form a trio of integrative interactomes with a high reproducibility across all the analyses. IBMP, despite belonging to the integrative–predictive class, is closer to such a trio than to other interactomes from the same class. Another trio of related interactomes, even if to a less extent, can be identified in DMND, MN and FP60. Among the four HTBP interactomes, HURI emerged as the most comparable to integrative and integrative–predictive interactomes.

While multiple efforts are underway on a medium/long time span to characterize a first consensus human reference interactome [11, 51], even including tissue specificity [52], guidelines are necessary to choose the most appropriate interactomes in computational analyses. Our analysis summarized the state of the art, characterizing the interactomes by a series of criteria that provide hints for interactome choice.

Considering the heterogeneity of designing principles and data sources in the process of interactome assembly, it is recommended to use multiple interactomes despite a novel interactome-based analysis. With this aim, it is possible to consider two data integration strategies. One may consider to repeat the analysis using different interactomes and, then, integrate the results. Another possibility is to integrate multiple interactomes into a meta-interactome and, then, use such unique interactome for the analysis. In both cases, criteria are needed for the choice of the interactomes.

A first criteria to bear in mind is interactome construction method. In general, interactomes that rely on experimentally verified interactions offer a more reliable body of knowledge, especially if interactions are scored on the basis of their reliability. However, integrative–predictive interactomes, like STRING, revealed interesting performances in disease gene prediction [15, 49]; in particular, text mining determined higher performances in such tasks, in which incorporating the existing knowledge matters [49]. On the other hand, interactomes that include predicted interactions tend to be denser than other interactomes, and, for example, when used to find subnetworks of ‘altered’ genes, this might lead to particularly dense subnetworks, where interpretation is not straightforward. The heterogeneity of designing principles suggests considering a representative interactome for each class.

The same goes for scale-freeness: considering the structural difference between a scale-free network and one that does not show this behavior, it would be interesting to consider a representative of each type.

Another criteria is interactome size (number of genes). The previous study of Huang *et al*. [15] showed that interactome size matters in disease gene prediction. We observed that bigger interactomes tend to capture a higher number of protein complexes as connected networks, while in the case of pathways the association is weaker. Therefore, it can be recommended to consider at least one of the large interactomes.

Our study provides catalogs of the level of coverage of protein complexes, pathways and PCT. This knowledge can be used as a criterion for choosing those interactomes that best match the molecular processes underlying the disease (or condition) under study.

The similarity among interactomes provides an interesting criterion that can be used as a guideline for the selection of interactomes; for example, one may choose a representative of each similarity cluster. Moreover, interactome choice can be inspired by the aggregate reproducibility index: indeed, high values indicate interactomes that are the most similar to all the others, while low values point to interactomes that are more specific.

In developing a meta-interactome, we could consider interactions recurring in more than one interactome. This approach was used to create a ‘parsimonious composite network’ with both high efficiency and performance in disease gene prediction [15]. In light of our results, this strategy seems particularly appropriate for interactomes that share some degree of similarity. However, if interactomes are likely to convey complementary information (e.g. HTBPs), one may also consider a union operation, which, especially in the case of experimentally verified interactions, should improve the coverage of the resulting meta-interactome. Therefore, a mixed strategy can be used, merging datasets that convey more reliable information and requiring link recurrence in those interactomes that mix heterogeneous resources and include predictions.

In conclusion, our comparison study was guided by some of the typical applications in which interactomes are used, related to protein complexes, molecular pathways, disease modules or markers, and the wide class of analyses involving topological properties of the network. Therefore, our results are influenced by the chosen analyses, even if we tried to be very exhaustive in terms of available analysis types.

Despite current limitations, interactome-based approaches represent a relevant tool to explain the complex (non-linear) relation between molecular alterations and pathological phenotypes. This knowledge is essential to translate gene-level findings into clinical practice, by means of more effective strategies for prevention, diagnosis and treatment [2].

**Key Points**

- A ‘consensus’ reference human interactome does not exist.
- Several interactomes, developed following different principles, are available and interactome choice matters greatly.
- Our study sheds light on heterogeneity, redundancy and specificity of interactomes from topological, biological and application perspectives.
- Interactomes are topologically heterogeneous, e.g. not all of them are scale free, and this might reflect differences in interactome reconstruction strategies.
- We provide catalogues of current coverage of protein complexes, pathways and pathway cross-talks.
- Almost all interactomes showed the best performance in disease gene prediction when considering cancer, rather than other diseases, possibly biased by the large amount of studies on cancer.
- The knowledge emerging from our analyses summarizes the current situation and can be useful to guide the choice of interactomes (singularly or in combination) in future applications.

## Supplementary Material

Supplementary_bbab153

## Notes

**Ettore Mosca** is a researcher of the Bioinformatics Lab at the Institute of Biomedical Technologies of the National Research Council (CNR-ITB). He is a biotechnologist and bioinformatician, and holds a PhD in Computer Science. His activities focus on network analysis, pathway analysis and multi-omics data integration in biology and biomedicine.

**Matteo Bersanelli** is a researcher at Humanitas University with background in applied mathematics, applied physics, big data and statistical learning. His applications mainly concern systems biology and systems medicine.

**Tommaso Matteuzzi** is a postdoc in Applied Physics at the Department of Physics and Astronomy of Bologna University. His research activity focuses on complex networks, biological network data integration and statistical modeling in biology and medicine.

**Noemi Di Nanni** is a research fellow of the Bioinformatics Lab at CNR-ITB. Her research interests include bioinformatics and computational biology.

**Gastone Castellani** is a full professor (Bologna University Department of Experimental, Diagnostic and Specialty Medicine) and director of the Galvani Center for Biocomplexity. He is an associate professor at Brown University and author of more than 200 peer-reviewed papers. His research interests are in machine learning, statistical learning and neural networks in biomedicine.

**Luciano Milanesi** is the coordinator of the CNR Interdepartmental Bioinformatics Research Network in Life Science, Medicine and ICT, and former research director at CNR-ITB. He is author of more than 400 peer-reviewed publications.

**Daniel Remondini** is a full professor (Bologna University Physics Department). His research interests are in machine learning and network theory applied to Systems Biology, and he is author of more than 100 peer-reviewed papers.
