# Supplementary material for: Characterization and comparison of gene-centered human interactomes
Source: Brief Bioinform. 2021 May 19;22(6):bbab153. doi: 10.1093/bib/bbab153 (PMC8574298; doi:10.1093/bib/bbab153)
Supplement: Supplementary_bbab153 [file supplementary_bbab153.docx]

## **SUPPLEMENTARY METHODS, TABLES AND FIGURES**

**Characterization and comparison of gene-centered human interactomes**

Ettore Mosca^1*^, Matteo Bersanelli^2,3^, Tommaso Matteuzzi^2^, Noemi Di Nanni^1^, Gastone Castellani^2^, Luciano Milanesi^1^ & Daniel Remondini^2*^

^1^Institute of Biomedical Technologies, National Research Council, Segrate (Milan), 20094, Italy

^2^Department of Physics and Astronomy, University of Bologna, & INFN, Bologna, 40127, Italy

^3^INFN, Bologna, 40127, Italy

*Correspondence:

Ettore Mosca

e-mail: [ettore.mosca@itb.cnr.it](mailto:ettore.mosca@itb.cnr.it)

telephone: +39 02 26422614

fax: +39 02 26422660

Daniel Remondini

e-mail: [daniel.remondini@unibo.it](mailto:daniel.remondini@unibo.it)

telephone: +39 051 2095127

**Supplementary Methods**

### Degree distribution fit…………………………………………………………………………………………………... 3

**Supplementary Tables**

[Table S1. Details about the interactomes considered in this study. 4](#_Toc67256309)

[Table S2. Main sources of interactions used in integrative interactomes. 6](#_Toc67256310)

[Table S3. Disease genes 7](#_Toc67256311)

[Table S4. Parameters (with errors) of the power law fit of the degree distribution and p-values of the fit computed with a semi-parametric bootstrap. 7](#_Toc67256312)

[Table S5. Correlation analysis. 8](#_Toc67256313)

[Table S6. CCFs of protein complexes. 8](#_Toc67256314)

[Table S7. CCFs of molecular pathways. 8](#_Toc67256315)

[Table S8. Pathway cross-talk. 8](#_Toc67256316)

[Table S9. Coverage of disease genes. 9](#_Toc67256317)

**Supplementary Figures**

[Figure S1. Scatter plots of mean distance (left) and mean transitivity, i.e. mean clustering coefficient (right) as a function of link density. 10](#_Toc63178357)

[Figure S2. Comparison of the mean distance (a) and mean transitivity (b) of each interactome (red) with a BA net (gray) with the same number of nodes and links. 11](#_Toc63178358)

[Figure S3. Degree distribution for each interactome. 12](#_Toc63178359)

[Figure S4. Recurrence of hubs. 13](#_Toc63178360)

[Figure S5. Heatmaps of pair-wise correlation of interactome centrality measures for each interactome. 14](#_Toc63178361)

[Figure S6. Correlation of centrality measures. 15](#_Toc63178362)

[Figure S7. Distribution of Spearman's correlation between the centrality measures across all interactomes pairs. 16](#_Toc63178363)

[Figure S8. Heatmaps of correlation of centrality measures on the overall common nodes subnetworks. 17](#_Toc63178364)

[Figure S9. Correlation of CCFs. 18](#_Toc63178365)

[Figure S10. Distribution of Spearman's correlation between the CCFs across interactomes. 19](#_Toc63178366)

[Figure S11. Distribution of Spearman's correlation between disease gene prioritization scores of different interactomes. 20](#_Toc63178367)

[Figure S12. Correlation and overlap matrices. 21](#_Toc63178368)

# **Supplementary Methods**

### ***Degree distribution fit***

Based on the hypothesis that the degree distribution follows a power law for degree greater than some threshold value *K_min_, we* jointly estimate *K_min_* and the power law exponent *α*. For a discrete power law, the maximum likelihood estimator (MLE) for *α*, fixed *K_min_*_,_ is given by:

$$\hat{\alpha}=1+n\left[ \sum_{i=1}^{n} \ln\frac{x_{i}}{K-0.5}] \right]^{-1}$$

where *x_i_* are the empirical data points. To estimate *K_min_* the MLE for α is computed varying *K* in the range [0, *max*(*Degree*)]. *K_min_* is the *K* which minimizes the Kolmogorov-Smirnoff (KS) distance between the data and fitted model cumulative density function. Since increasing *K* the number of data points available for the fit of *α decreases* , a minimum number of points in the tail is required for not to discard the power law hypothesis.

Goodness-of-fit is assessed by a semi-parametric bootstrap procedure. A fixed number, *N_bs_* of synthetic distributions is generated in the following way:

- for degree lower than the estimated *K_min_*, points are bootstrapped from the empirical data,
- for degree higher than the *K_min_*, points are sampled from the best fit power law distribution

Than, for each synthetic distribution fit, the KS statistics is computed. A p-value is defined as the fraction of times the KS of the fit of the synthetic distributions is greater than that for the empirical data fit. Therefore, a high p-value indicates that the power law fits real data as good as synthetic data and cannot be rejected. Finally, uncertainties on *K_min_* and α are estimated sampling (with replacement) from the original data set and re-estimating the parameters.

| **Acronym** | **version** | **source** | **Initial**  **identifier** | **Links**  **(I)** | **Vertices**  **(I)** | **Links**  **(M)** | **Vertices**  **(M)** | **Links**  **(F)** | **Vertices**  **(F)** | **Links**  **(LCC)** | **Vertices**  **(LCC)** | **LCC**  **%** | **M-LCC** | **M-LCC**  **(%)** |
| --- | --- | --- | --- | --- | --- | --- | --- | --- | --- | --- | --- | --- | --- | --- |
| BX | v4a | https://bioplex.hms.harvard.edu/data/BioPlex_interactionList_v4a.tsv | Entrez Gene | 56'533 | 10'961 | 10'940 | 56'440 | NA | NA | 10'880 | 56'401 | 100 | 39 | 0.069 |
| CF | publication | Supplementary Table 2. PPIs Interologs and Corum | Ensemble gene | 16'655 | 3'464 | 15'635 | 3'349 | NA | NA | 15'513 | 3'191 | 95 | 158 | 4.718 |
| FP60 | publication | Supplementary Data 5. Fp60 network: predicted interactions with estimated FDR of 60%. | Uniprot | 250'498 | 9'456 | 258'337 | 10'532 | NA | NA | 258'107 | 10'403 | 99 | 129 | 1.225 |
| DMND | publication | Supplementary File S1 Data | Entrez Gene | 141'296 | 13'460 | 141'020 | 13'372 | NA | NA | 138'045 | 13'244 | 99 | 128 | 0.957 |
| HN | Apr-19 | http://hint.yulab.org/download | Uniprot | 386'348 | 13'686 | 169'679 | 14'643 | NA | NA | 164'255 | 14'372 | 98 | 271 | 1.851 |
| HP | v2.2 | http://cbdm-01.zdv.uni-mainz.de/~mschaefer/hippie/download.php | Entrez Gene | 410'618 | 18'166 | 408'895 | 18'067 | NA | NA | 404'020 | 18'038 | 100 | 29 | 0.161 |
| HURI | HI-I, HI-II, Lit-BM | http://interactome.baderlab.org/download | Ensembl transcript | 36'035 | 8'481 | 36'020 | 8'471 | NA | NA | 27'084 | 8'029 | 95 | 442 | 5.218 |
| MN | publication | http://homes.gersteinlab.org/Khurana-PLoSCompBio-2013 | Gene symbol | 109'598 | 14'445 | 105'617 | 13'433 | NA | NA | 105'573 | 13'387 | 100 | 46 | 0.342 |
| NCBI | 26-Feb-19 | ftp://ftp.ncbi.nih.gov/gene/GeneRIF | Entrez Gene | 347'140 | 20'818 | 330'592 | 17'682 | NA | NA | 326'859 | 17'655 | 100 | 27 | 0.153 |
| QU | IM-24272 | http://www.imexconsortium.org | Uniprot | 14'988 | 4'664 | 14'988 | 4'384 | NA | NA | 14'696 | 4'379 | 100 | 5 | 0.114 |
| ST04 | v11 | https://string-db.org | Ensemble protein | 11'759'454 | 19'354 | 11'494'006 | 18'920 | 981'463 | 15'958 | 490'587 | 15'800 | 99 | 158 | 0.835 |
| S04T | v11 | https://string-db.org | Ensemble protein | 11'759'454 | 19'354 | 11'494'006 | 18'920 | 1'972'154 | 18'863 | 986'054 | 18'863 | 100 | 0 | 0.000 |
| S07 | v11 | https://string-db.org | Ensemble protein | 11'759'454 | 19'354 | 11'494'006 | 18'920 | 714'690 | 13'083 | 357'054 | 12'747 | 97 | 336 | 1.776 |
| S07T | v11 | https://string-db.org | Ensemble protein | 11'759'454 | 19'354 | 11'494'006 | 18'920 | 834'359 | 16'994 | 417'012 | 16'721 | 98 | 273 | 1.443 |
| IR | 15 | http://irefindex.org/wiki/index.php?title=iRefIndex | iRefIndex icrogid | 709'259 | 27'303 | 532'911 | 17'553 | NA | NA | 476'437 | 17'522 | 100 | 31 | 0.177 |
| BN | guildify 2.0 | http://aleph.upf.edu/guildify2/downloads | Entrez Gene | 339'722 | 13'283 | 339'718 | 13'282 | NA | NA | 339'698 | 13'246 | 100 | 36 | 0.271 |
| IBMP | 2016_09_12 | https://www.intomics.com/inbio/map.html#downloads | Uniprot | 625'641 | 17'653 | 653'510 | 17'466 | NA | NA | 652'636 | 17'458 | 100 | 8 | 0.046 |
| CP | guildify 2.0 | http://aleph.upf.edu/guildify2/downloads | Entrez Gene | 273'067 | 16'095 | 273'025 | 16'089 | NA | NA | 273'005 | 16'066 | 100 | 23 | 0.143 |
| INCT | 2019_07_03 | ftp://ftp.ebi.ac.uk/pub/databases/intact/2019-05-02/psimitab/ | Uniprot | 231'952 | 25'644 | 196'458 | 15'586 | NA | NA | 174'388 | 15'539 | 100 | 47 | 0.302 |

**Table S1. Details about the interactomes considered in this study.**

I: Initial; M: after mapping; F: final; LCC: largest connected component; M-LCC: number of vertices not belonging to the LCC; M-LCC (%): percentage of M vertices not included in the LCC.

|  | **BN** | **CP** | **FP60** | **DMND** | **HINT** | **HP** | **IBMP** | **IR** | **MN** | **NCBI** | **ST*** |
| --- | --- | --- | --- | --- | --- | --- | --- | --- | --- | --- | --- |
|  | *Guildify 2.0 Supplementary File* | *website* | *Not found* | *publication* | *website* | *publication* | *publication supplementary data S2* | *website* | *publication* | *website* | *Last publication + website* |
| **BHF_UCL** |  |  |  |  |  |  |  | X |  |  |  |
| **BIND** |  | X |  |  |  | X | X | X |  | X |  |
| **BIGG** |  |  |  | X |  |  |  |  |  |  |  |
| **BIOCARTA** |  | X |  |  |  |  |  |  |  |  |  |
| **BIOCYC** |  |  |  |  |  |  |  |  |  |  | X |
| **BIOGRID** | X | X |  | X | X | X | X | X | X | X | X |
| **Chembl** |  | X |  |  |  |  |  |  |  |  |  |
| **CORUM** |  | X |  | X |  |  |  | X |  |  |  |
| **DIP** | X | X |  |  | X | X | X | X |  |  | X |
| **DrugBank** |  | X |  |  |  |  |  |  |  |  |  |
| **EHMN** |  | X |  |  |  |  |  |  |  |  |  |
| **EcoCyc** |  |  |  |  |  |  |  |  |  | X |  |
| **ENCODE** |  |  |  |  |  |  |  |  | X |  |  |
| **Gene Ontology** |  |  |  |  |  |  |  |  |  |  | X |
| **HIV** |  |  |  |  |  |  |  |  |  | X |  |
| **HIPPIE** | X |  |  |  |  |  |  |  |  |  |  |
| **HPIDB** |  |  |  |  |  |  |  | X |  |  |  |
| **HPRD** |  | X |  | X | X | X |  | X |  | X |  |
| **HumanCyc** |  | X |  |  |  |  |  |  |  |  |  |
| **I2D_IMEx** |  |  |  |  |  |  |  | X |  |  |  |
| **IMEx** |  |  |  |  |  |  |  |  |  |  | X |
| **InnateDB** |  | X |  |  |  |  |  | X |  |  |  |
| **INOH** |  | X |  |  |  |  |  |  |  |  |  |
| **IntAct** | X | X |  | X | X | X | X | X |  |  | X |
| **iRefWeb** | X |  |  |  | X |  |  |  |  |  |  |
| **KEGG** |  | X |  | X |  |  |  |  | X |  | X |
| **MatrixDB** |  | X |  |  |  |  | X | X |  |  |  |
| **MINT** |  | X |  | X | X | X |  | X |  |  | X |
| **MIPS** |  | X |  |  | X | X |  |  |  |  |  |
| **Molcon** |  |  |  |  |  |  |  | X |  |  |  |
| **MPact** |  |  |  |  |  |  |  | X |  |  |  |
| **MPIDB** |  |  |  |  |  |  |  | X |  |  |  |
| **MPPI** |  | X |  |  |  |  |  | X |  |  |  |
| **NetPath** |  | X |  |  |  |  | X |  | X |  |  |
| **PDB** |  | X |  |  | X |  |  |  |  |  | X |
| **PDZBase** |  | X |  |  |  |  |  |  |  |  |  |
| **PhosphoPOINT** |  | X |  |  |  |  |  |  |  |  |  |
| **PhosphositePlus** |  | X |  | X |  |  |  |  |  |  |  |
| **PID** |  | X |  |  |  |  |  |  |  |  | X |
| **PIG** |  | X |  |  |  |  |  |  |  |  |  |
| **PINdb** |  |  |  |  |  |  |  |  |  |  |  |
| **QuickGO** |  |  |  |  |  |  |  | X |  |  |  |
| **REACTOME** |  | X |  |  |  |  | X | X | X |  | X |
| **SIGNALINK** |  | X |  |  |  |  |  |  | X |  |  |
| **SIMAP** |  |  |  |  |  |  |  |  |  |  | X |
| **SPIKE** |  | X |  |  |  |  |  |  |  |  |  |
| **TRANSFAC** |  |  |  | X |  |  |  |  |  |  |  |
| **UNIPROTPP** |  |  |  |  |  |  |  | X |  |  |  |
| **Vinayagam et al. Science Signaling 2011** |  |  |  | X |  |  |  |  |  |  |  |
| **VIRUSHOST** |  |  |  |  |  |  |  | X |  |  |  |
| **Wikipathways** |  | X |  |  |  |  | X |  |  |  |  |

**Table S2. Main sources of interactions used in integrative interactomes.**

**Table S3. Disease genes**

Available at URL <https://github.com/emosca-cnr/interactome-comparison-data>

| **interactome** | **p-val** | **Kmin** | **Kmin error** | **alpha** | **alpha error** | **Alternatives** |
| --- | --- | --- | --- | --- | --- | --- |
| **BX** | **0.78** | 16 | 1 | 2.88 | 0.07 | none |
| **CF** | **0.59** | 15 | 2 | 2.62 | 0.1 | none |
| **HURI** | **0.84** | 26 | 8 | 2.68 | 0.2 | exponential |
| **QU** | **0.7** | 16 | 5 | 2.6 | 0.18 | exponential |
| **DMND** | **0.5** | 15 | 80 | 2.01 | 1.35 | exponential |
| **HN** | **0.33** | 129 | 28 | 3.75 | 0.38 | exponential |
| **HP** | **0.18** | 245 | 70 | 3.11 | 0.3 | exponential |
| **IR** | **0.17** | 299 | 27 | 4 | 0.23 | none |
| **NCBI** | 0.1 | 75 | 42 | 2.68 | 0.17 | exponential |
| **FP60** | 0.01 | 194 | 53 | 3.13 | 0.33 | none |
| **MN** | **0.94** | 39 | 6 | 2.42 | 0.05 | exponential |
| **S04** | **0.5** | 405 | 60 | 6.01 | 0.58 | exponential |
| **S04T** | **0.69** | 415 | 54 | 4.74 | 0.31 | exponential |
| **S07** | 0 | 221 | 29 | 4.95 | 0.26 | none |
| **S07T** | 0.01 | 297 | 32 | 5.3 | 0.31 | exponential |
| **BN** | 0 | 7 | 1 | 1.66 | 0.01 | exponential |
| **CP** | **0.48** | 232 | 57 | 3.58 | 0.34 | exponential |
| **IBMP** | 0 | 186 | 63 | 2.8 | 0.15 | exponential |
| **INTC** | 0 | 49 | 17 | 2.65 | 0.13 | exponential |

**Table S4. Parameters (with errors) of the power law fit of the degree distribution and p-values of the fit computed with a semi-parametric bootstrap.**

Parameters are in bold if the corresponding p-value > 0.1, which means that the fit of empirical data was better than that of the semi-parametric bootstrap in at least 10% of the iterations indicating that the power law hypothesis cannot be rejected. Kmin and α are, respectively, the onset threshold and the exponent of the power law fit; parameter values are reported with errors determined with a bootstrap procedure (described in methods). The column Alternatives indicates if exponential and lognormal distributions fit data better than power law.

**Table S5. Correlation analysis.**

Available at URL https://github.com/emosca-cnr/interactome-comparison-data

**Table S6. CCFs of protein complexes.**

Available at URL https://github.com/emosca-cnr/interactome-comparison-data

**Table S7. CCFs of molecular pathways.**

Available at URL https://github.com/emosca-cnr/interactome-comparison-data

**Table S8. Pathway cross-talk.**

Available at URL https://github.com/emosca-cnr/interactome-comparison-data

|  | **Cosmic**  **(237)** | | **Rheumatoid Arthritis**  **(174)** | | **Parkinson Disease**  **(109)** | | **Autistic Disorder**  **(241)** | | **Ataxias, Hereditary**  **(155)** | |
| --- | --- | --- | --- | --- | --- | --- | --- | --- | --- | --- |
|  | # | % | # | % | **#** | % | # | % | # | % |
| **S04T** | 237 | **100** | 174 | **100** | 107 | **98.2** | 238 | **98.8** | 153 | **98.7** |
| **IBMP** | 237 | **100** | 173 | **99.4** | 107 | **98.2** | 237 | **98.3** | 152 | **98.1** |
| **IR** | 237 | **100** | 173 | **99.4** | 107 | **98.2** | 235 | **97.5** | 150 | **96.8** |
| **HP** | 236 | **99.6** | 170 | **97.7** | 108 | **99.1** | 235 | **97.5** | 152 | **98.1** |
| **S07T** | 236 | **99.6** | 171 | **98.3** | 106 | **97.2** | 237 | **98.3** | 151 | **97.4** |
| **NCBI** | 237 | **100** | 169 | **97.1** | 107 | **98.2** | 233 | **96.7** | 150 | **96.8** |
| **CP** | 237 | **100** | 170 | **97.7** | 106 | **97.2** | 230 | **95.4** | 149 | **96.1** |
| **S04** | 236 | **99.6** | 168 | **96.6** | 103 | **94.5** | 228 | **94.6** | 141 | **91** |
| **INCT** | 234 | **98.7** | 165 | **94.8** | 104 | **95.4** | 214 | **88.8** | 144 | **92.9** |
| HINT | 233 | **98.3** | 162 | **93.1** | 103 | **94.5** | 205 | 85.1 | 139 | 89.7 |
| DMND | 233 | **98.3** | 159 | **91.4** | 102 | **93.6** | 219 | **90.9** | 128 | 82.6 |
| MN | 232 | **97.9** | 159 | **91.4** | 97 | 89 | 211 | 87.6 | 130 | 83.9 |
| S07 | 229 | **96.6** | 156 | 89.7 | 93 | 85.3 | 204 | 84.6 | 125 | 80.6 |
| BN | 232 | **97.9** | 143 | 82.2 | 96 | 88.1 | 201 | 83.4 | 131 | 84.5 |
| FP60 | 227 | **95.8** | 147 | 84.5 | 90 | 82.6 | 189 | 78.4 | 111 | 71.6 |
| HURI | 206 | 86.9 | 98 | 56.3 | 74 | 67.9 | 132 | 54.8 | 75 | 48.4 |
| BP | 177 | 74.7 | 95 | 54.6 | 72 | 66.1 | 119 | 49.4 | 103 | 66.5 |
| QU | 106 | 44.7 | 29 | 16.7 | 34 | 31.2 | 42 | 17.4 | 55 | 35.5 |
| CF | 74 | 31.2 | 24 | 13.8 | 28 | 25.7 | 24 | 10 | 44 | 28.4 |

**Table S9. Coverage of disease genes.**

Number (#) and percentage (%) of disease associated genes available in interactomes, out of the total (below disease name); interactomes are sorted in decreasing order of coverage of disease genes; boldface indicates values beyond 90% and interactomes that reach 90% in all 5 diseases.


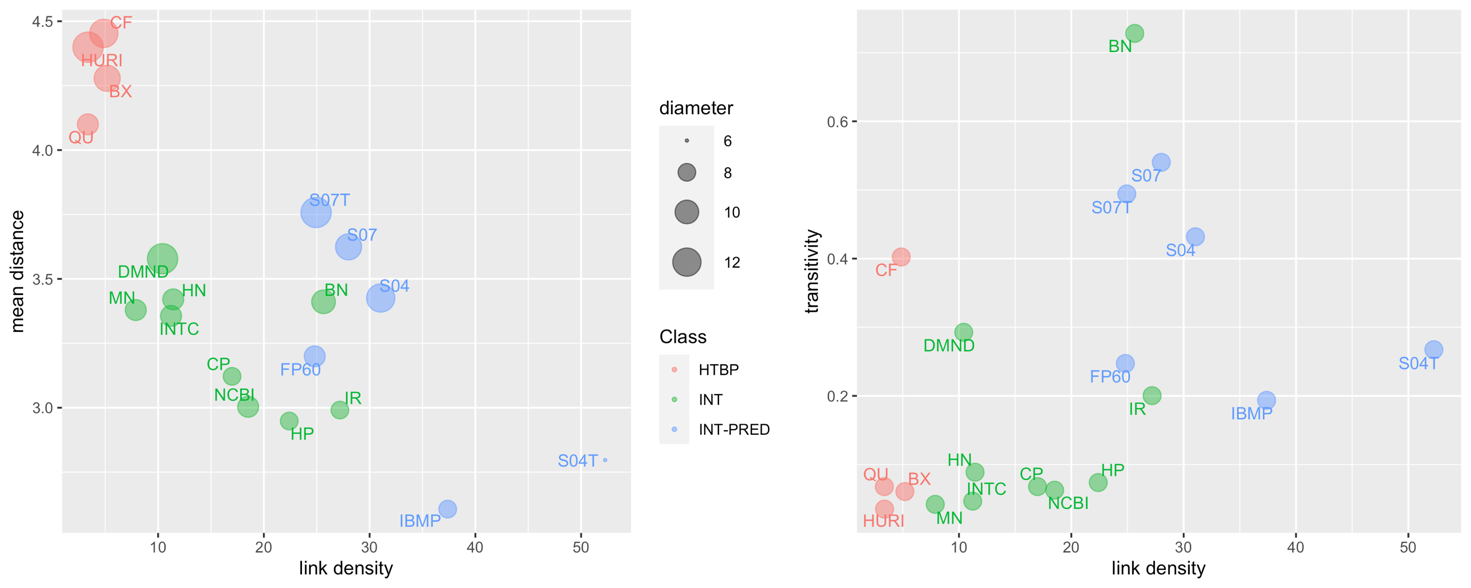


**Figure S1. Scatter plots of mean distance (left) and mean transitivity, i.e. mean clustering coefficient (right) as a function of link density.**

In the panel on the left dot size is proportional to the network diameter. Overall, lower density reflects higher mean distance and lower transitivity. In the left panel we can identify two groups: one comprises interactomes with link density < 25 (link per node) and a similar mean transitivity (~0.05), the other comprises denser interactomes with transitivity spanning a much larger range (~0.2 – ~0.75).


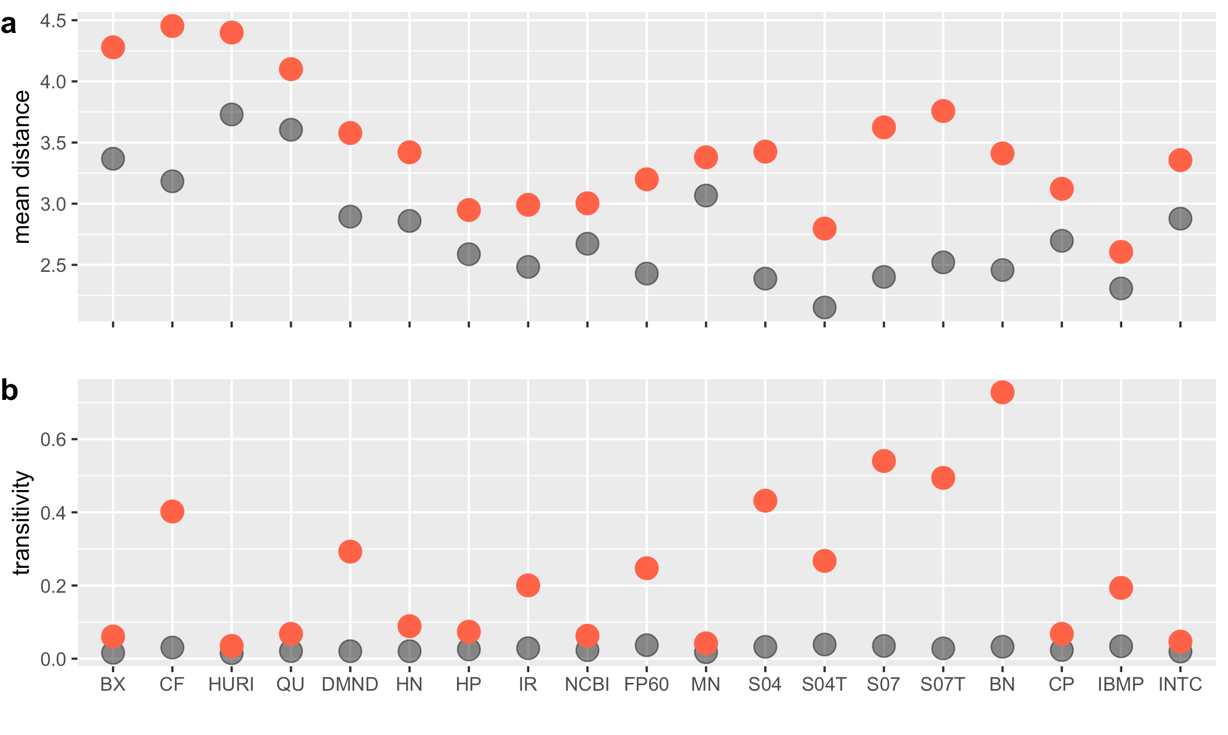


**Figure S2. Comparison of the mean distance (a) and mean transitivity (b) of each interactome (red) with a BA net (gray) with the same number of nodes and links.**

For each BA net the quantities are averaged over 10 different instances of the net. The variance is contained in the dot size and is generally small with respect to the difference with the corresponding interactome.


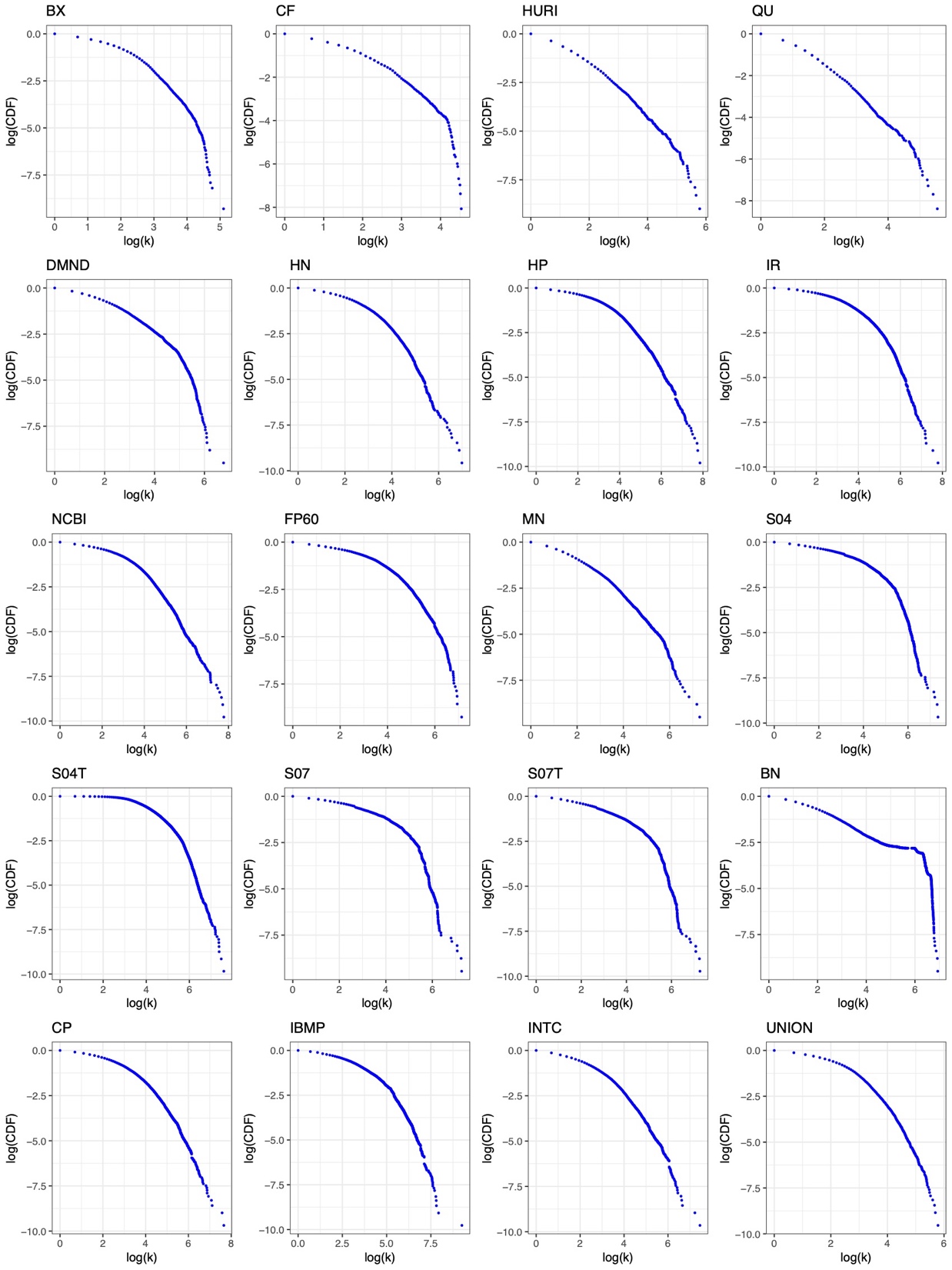


**Figure S3. Degree distribution for each interactome.**

Log-Log scale plot. k: degree, CDF: cumulative distribution function.


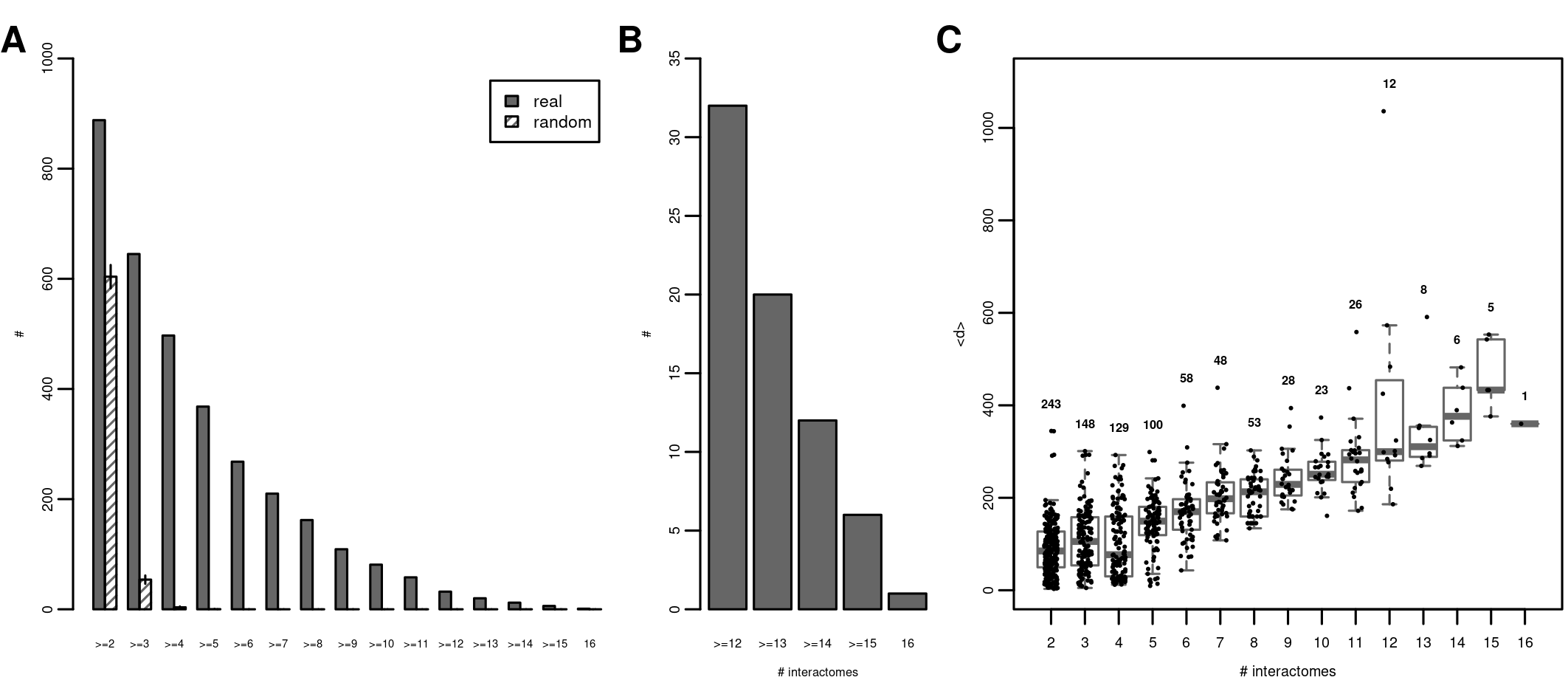


**Figure S4. Recurrence of hubs.**

**(A)** Number of genes in the top 2 percentiles of the degree distribution (hubs) of each interactome that are shared by 2 or more interactomes (real) or by permutations of the same degree distributions (random). **(B)** Hubs shared by at least 12 interactomes. **(C)** Degree of hubs and their recurrence in interactomes.

**Figure S5. Heatmaps of pair-wise correlation of interactome centrality measures for each interactome.**

The general pattern is preserved for all interactome. In particular, degree, betweenness and closeness have correlation greater than 0.8 on the whole set of the interactomes. Spectral centrality, even if less correlated with the others (~0.5), preserve the same patterns across all interactomes.


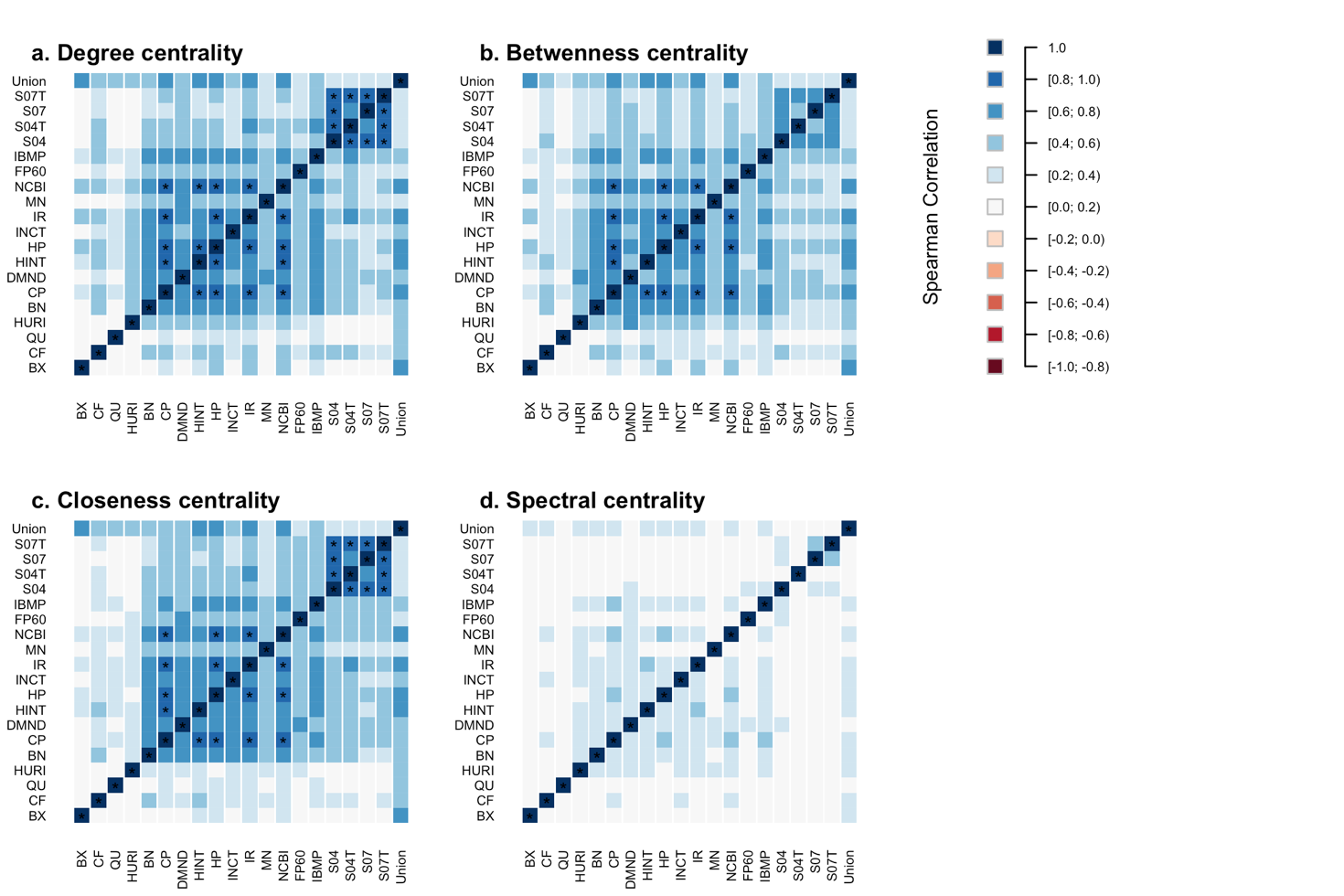


**Figure S6. Correlation of centrality measures.**

Correlation of each interactome centrality with the network resulting from the union of the four HTBP interactome is included. (*) correlation higher >= 0.8.


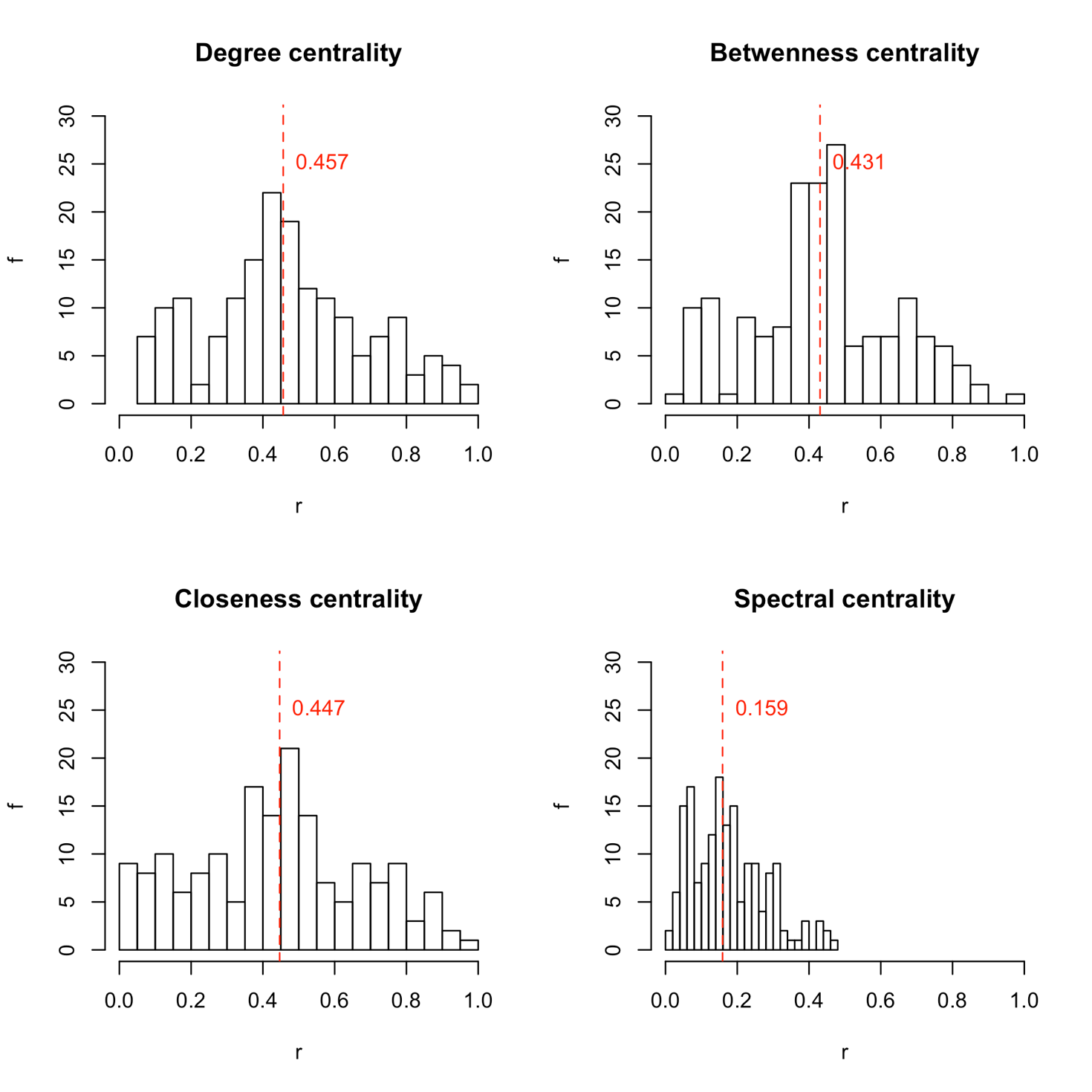


**Figure S7. Distribution of Spearman's correlation between the centrality measures across all interactomes pairs.**

The red line indicates the median.


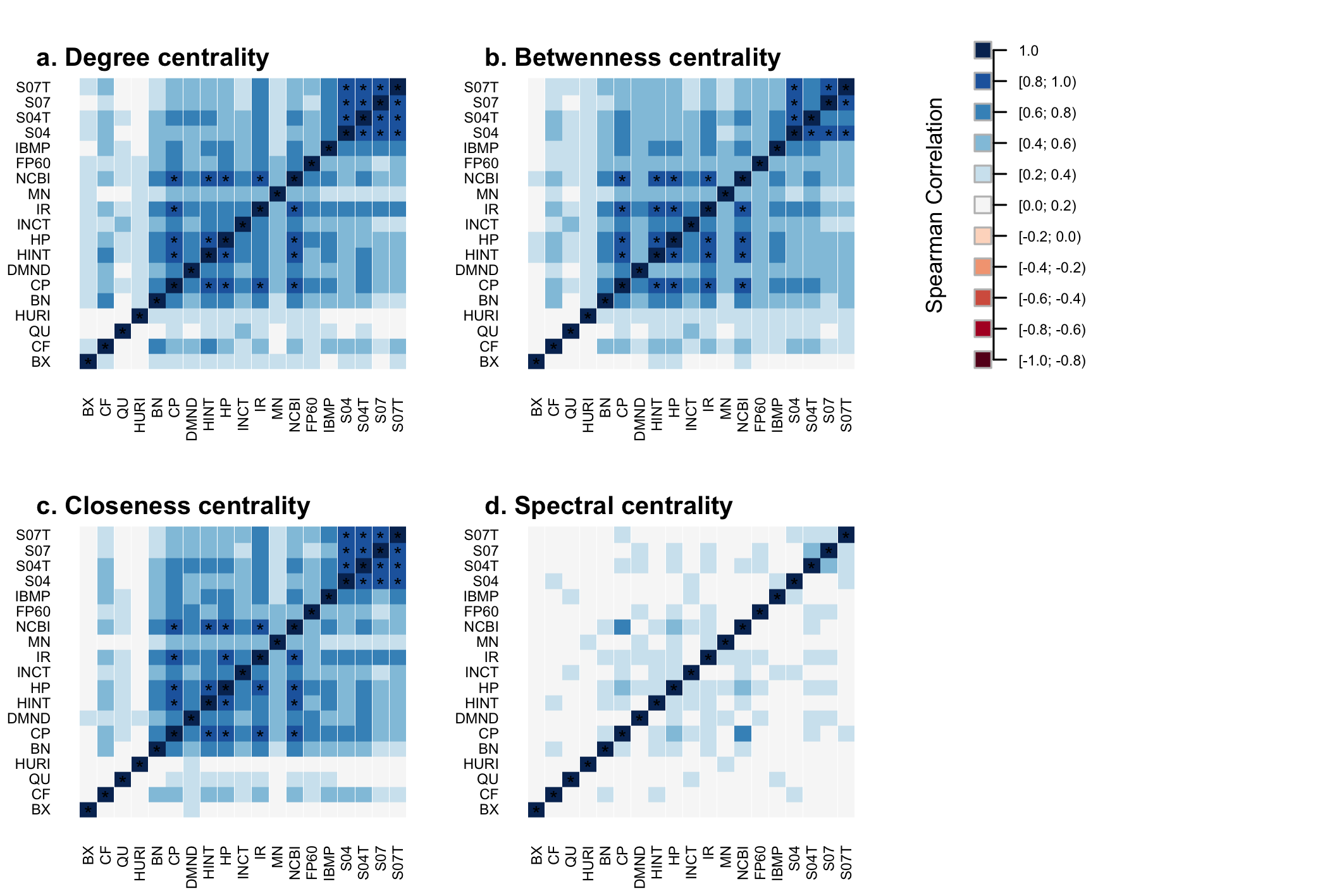


**Figure S8. Heatmaps of correlation of centrality measures on the overall common nodes subnetworks.**

For each interactome, the subnetwork induced by the 1021 genes in common to all interactomes was built. In figure is shown the correlation between each pair of centrality measure vectors carried out on these subnetworks. (*) correlation >= 0.8.


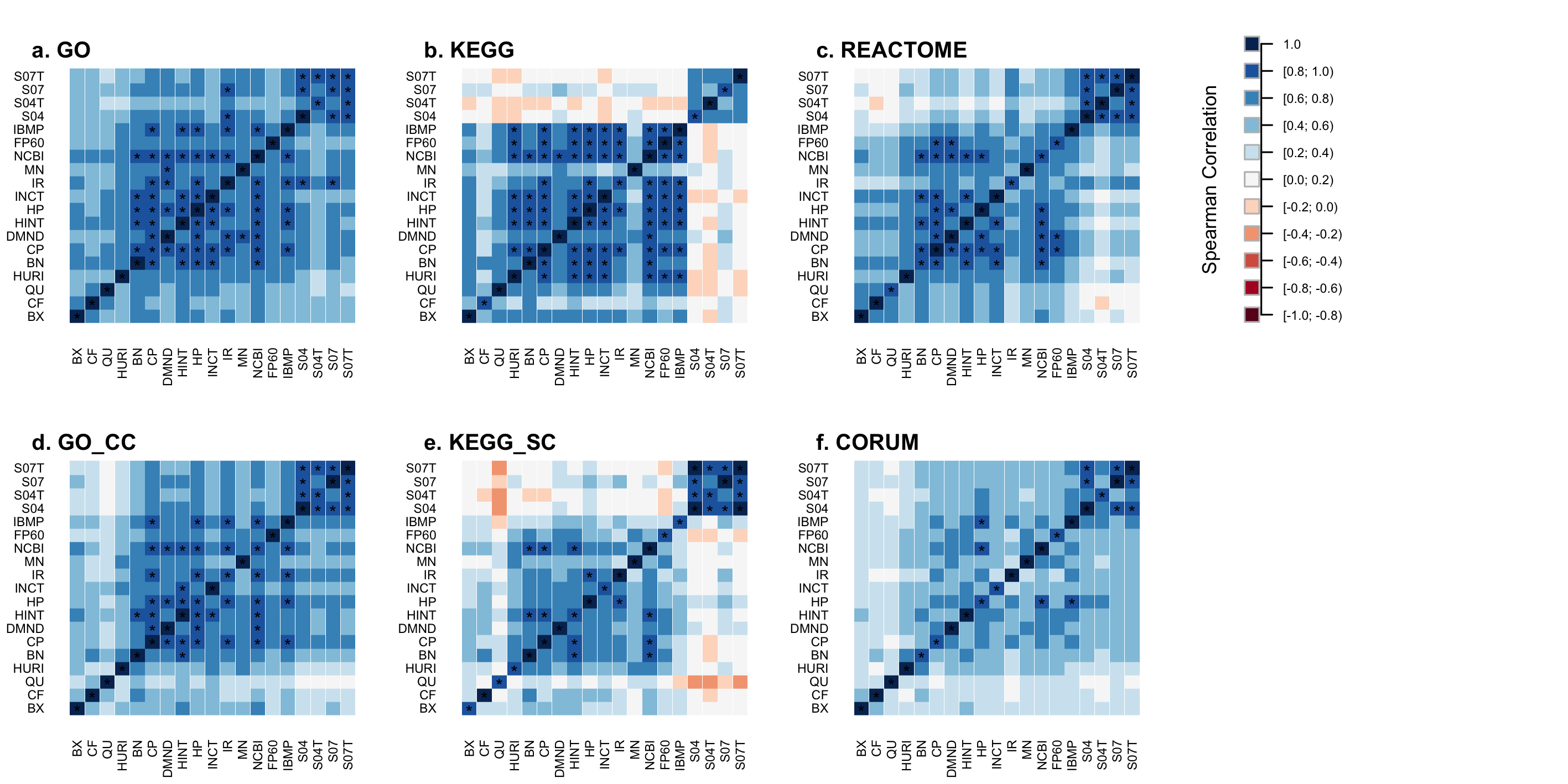


**Figure S9. Correlation of CCFs.**

**(a-c)** Molecular pathways. **(d-f)** Protein complexes.


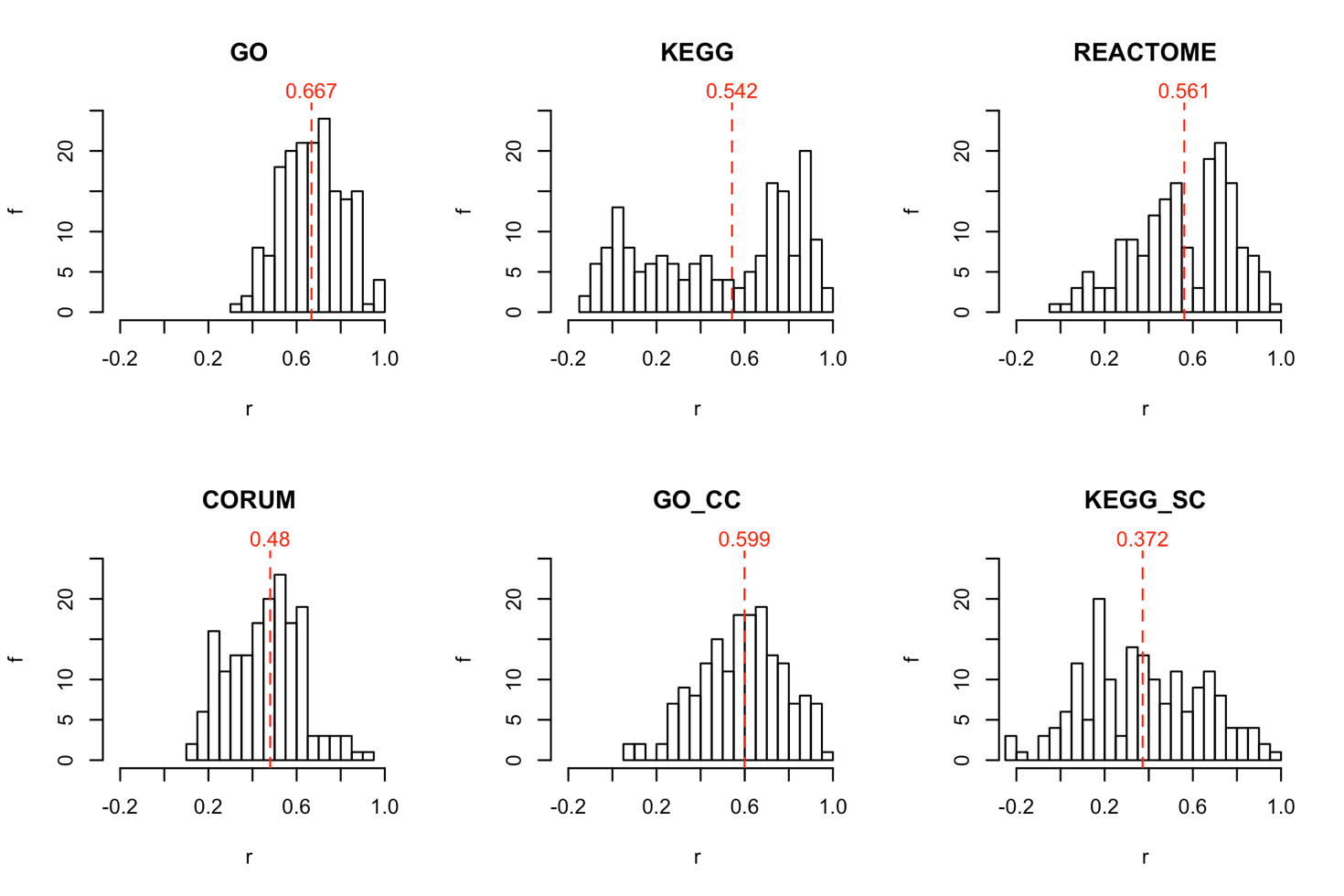


**Figure S10. Distribution of Spearman's correlation between the CCFs across interactomes.**

Top: pathways; bottom: protein complexes. The red line indicates the median.


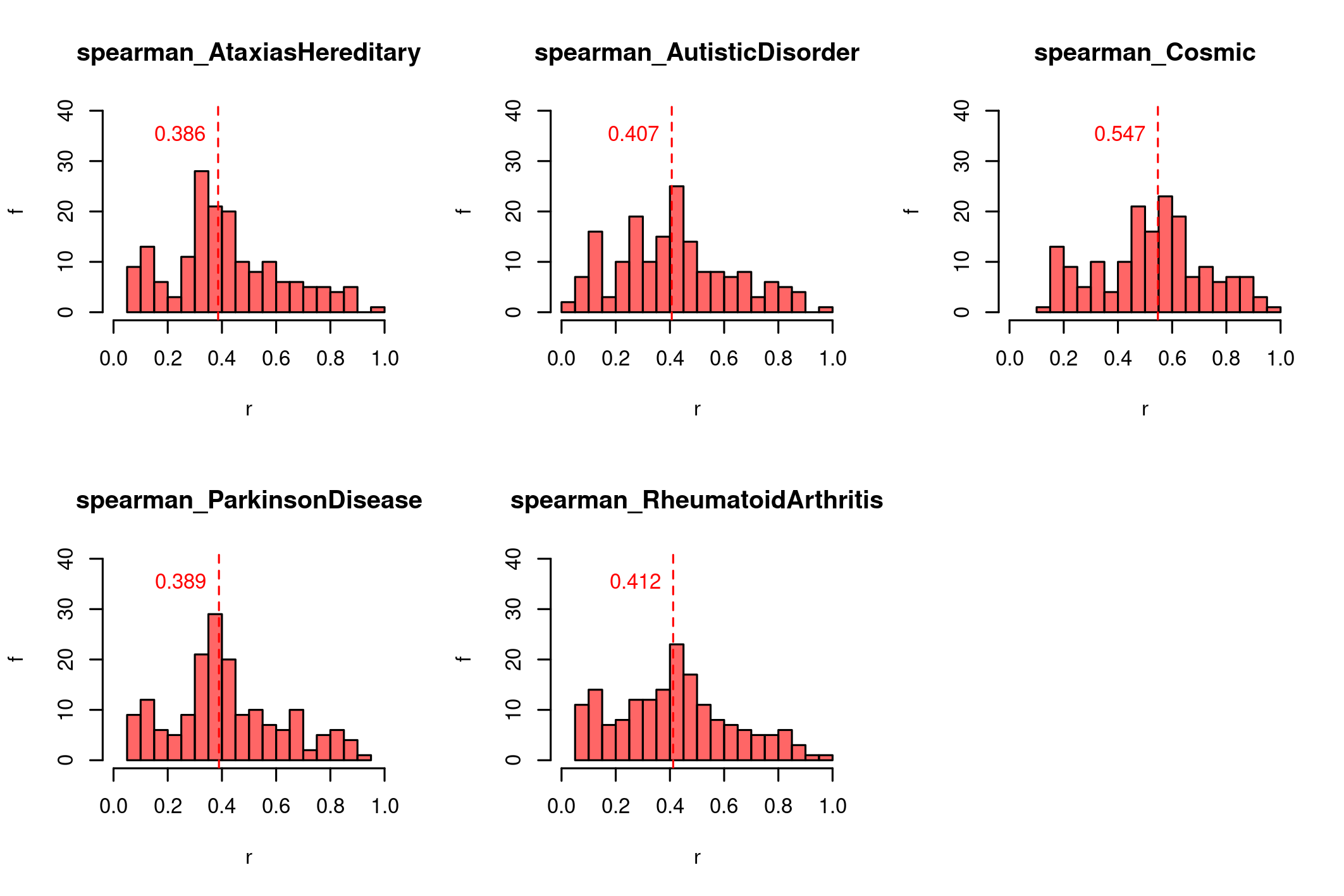


**Figure S11. Distribution of Spearman's correlation between disease gene prioritization scores of different interactomes.**

Vertical lines indicate medians.


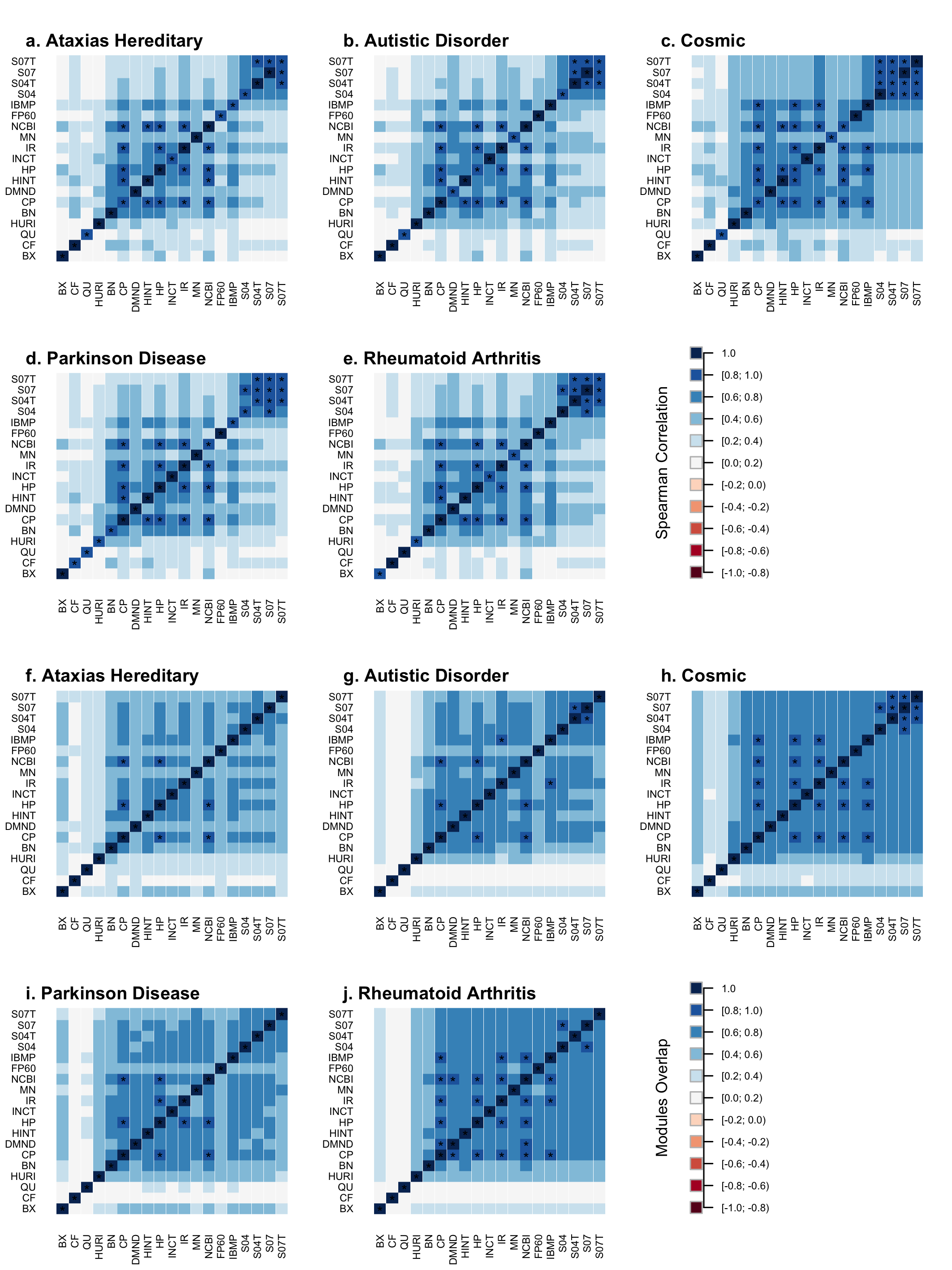


**Figure S12. Correlation and overlap matrices.**
